# Proteomic Profiling in *Drosophila* Reveals Potential Dube3a Regulation of the Actin Cytoskeleton and Neuronal Homeostasis

**DOI:** 10.1371/journal.pone.0061952

**Published:** 2013-04-23

**Authors:** Laura Jensen, M. Febin Farook, Lawrence T. Reiter

**Affiliations:** 1 Department of Neurology, University of Tennessee Health Science Center, Memphis, Tennessee, United States of America; 2 Department of Pediatrics, University of Tennessee Health Science Center, Memphis, Tennessee, United States of America; 3 Department of Anatomy and Neurobiology, University of Tennessee Health Science Center, Memphis, Tennessee, United States of America; Louisiana State University Health Sciences Center, United States of America

## Abstract

The molecular defects associated with Angelman syndrome (AS) and 15q duplication autism are directly correlated to expression levels of the E3 ubiquitin ligase protein UBE3A. Here we used *Drosophila melanogaster* to screen for the targets of this ubiquitin ligase under conditions of both decreased (as in AS) or increased (as in dup(15)) levels of the fly Dube3a or human UBE3A proteins. Using liquid phase isoelectric focusing of proteins from whole fly head extracts we identified a total of 50 proteins that show changes in protein, and in some cases transcriptional levels, when Dube3a fluctuates. We analyzed head extracts from cytoplasmic, nuclear and membrane fractions for Dube3a regulated proteins. Our results indicate that Dube3a is involved in the regulation of cellular functions related to ATP synthesis/metabolism, actin cytoskeletal integrity, both catabolism and carbohydrate metabolism as well as nervous system development and function. Sixty-two percent of the proteins were >50% identical to homologous human proteins and 8 have previously be shown to be ubiquitinated in the fly nervous system. Eight proteins may be regulated by Dube3a at the transcript level through the transcriptional co-activation function of Dube3a. We investigated one autism-associated protein, ATPα, and found that it can be ubiquitinated in a Dube3a dependent manner. We also found that Dube3a mutants have significantly less filamentous actin than wild type larvae consistent with the identification of actin targets regulated by Dube3a. The identification of UBE3A targets is the first step in unraveling the molecular etiology of AS and duplication 15q autism.

## Introduction

Angelman syndrome (AS) is a rare and severely debilitating neurological disorder with an incidence of ∼1/10–20,000 children [1]. Characteristics of AS include severe intellectual disability, inappropriate laughter, stereotypical hand movements, lack of speech, seizures and ataxic gait (Online Mendelian Inheritance in Man #105830) [2]. The underlying genetic cause for these features is the loss of expression of an E3 ubiquitin ligase protein called UBE3A located on chromosome 15q11.2. Although there are many ways to inherit AS due to the complexity of *UBE3A* transcriptional regulation and imprinting [3], the loss of UBE3A protein expression in the brain is the underlying molecular lesion resulting in the AS phenotype [4], [5]. While maternal deletions of 15q11.2-q13 cause AS, maternal duplications of this region consistently result in an autism phenotype (reviewed in [6]). A key indicator that the maternally expressed *UBE3A* gene is primarily responsible for the autism phenotype in individuals with 15q duplication is the fact that paternal duplications, where the *UBE3A* gene is silent on the duplicated allele, can often be non-pathogenic or do not involve a clear ASD phenotype [7]–[10]. A recent mouse model of duplication 15q autism confirms that elevated dosage of the *UBE3A* gene is sufficient to produce autism-like behaviors in a mouse model of 15q duplication syndrome lending additional support to the hypothesis that elevation of UBE3A levels in the brain is the primary cause of autism in duplication 15q syndrome [11].

The UBE3A protein is an E3 ubiquitin ligase (also known as E6-AP, for E6 associated protein [12]) that transfers a single ubiquitin moiety from its E2 partner to a protein target. These proteins are subsequently targeted for degradation, cellular trafficking, phosphorylation or subjected to site specific cleavage depending on the site of ubiquitination and the degree of mono vs polyubiquitination involved or even the chain of the lysine residue being modified [13]. Furthermore, this process of localized regulation via the ubiquitin proteasome pathway appears to play a major role not only in neurodegenerative disorders such as Alzheimer’s and Huntington’s disease, but also in the basic process of synaptic plasticity in normal neurons [14], [15]. A growing body of evidence from studies of proteins expressed in the post-synaptic density, and the turn over of glutamate receptors in cultured neurons, indicates that experience dependent synaptic plasticity requires ubiquitination and the ubiquitin (Ub)-dependent degradation of proteins at the synapse [16], [17]. In fact, *Ube3a* deficient mice show defects specifically in experience dependent plasticity [15], [18]. In addition, both *Ube3a* deficient and *Ube3a* over-expressing animals have defects in glutamatergic synaptic transmission [11], [15]. Although several mouse knock out models of AS have been made which recapitulate both the loss and gain of *Ube3a* expression in the brain [11], [15], [19]–[21], only a small handful of protein substrates of Ube3a that could ultimately be responsible for the neurological phenotypes observed in mice have been identified [22], [23], [24].

Our laboratory has demonstrated that over-expression of human UBE3A protein in the *Drosophila* central nervous system is an effective way to screen for putative UBE3A targets. Using this methodology in combination with 2-dimensional gel (2-D gel) electrophoresis we identified a protein that is a master regulator of actin cytoskeleton remodeling (the Rho-GEF ECT2) which physically interacts with both human and fly Dube3a proteins *in vivo* and may influence phenotypes related to actin cytoskeletal remodeling [23]. We also used this screening method to identify a transcriptionally regulated Dube3a target that is a key regulator of dopamine synthesis in flies and humans, the monoamine pathway rate limiting enzyme GTP Cyclohydrolase I [25]. Considering the problems of dynamic range when performing 2-D gel analysis used in previous studies to identify differentially regulated proteins we have chosen to use preparative liquid phase isoelectric focusing (IEF) for a more comprehensive screen of Dube3a regulated proteins in fly heads [26].

Here we utilize this more quantitative and reproducible preparative liquid phase isoelectric focusing (IEF) cell in combination with traditional 1-D denaturing polyacrylamide gel electrophoresis (SDS-PAGE) to separate proteins by molecular weight and quantify changes in protein levels dependent on fluctuations in Dube3a levels. This method allows us to resolve aliquots from the same fractions repeatedly until the pH comparisons are complete and the appropriate protein bands have been excised from the gels for mass spectrometry protein identification.

We directly compared nuclear, cytoplasmic and membrane extracts from *Drosophila melanogaster* heads expressing either no Dube3a protein (*Dube3a^15b^*) or high levels of human or fly UBE3A proteins. By analyzing three cellular compartments we were not only able to identify proteins regulated by Dube3a involved in transcription or extracellular cellular signaling, but were also able to identify proteins that shifted from one cellular compartment to another in a Dube3a, and thus possibly ubiquitination, dependent manner. Using this approach we identified 50 proteins potentially regulated by UBE3A and implicating Dube3a involvement in neuronal homeostasis, ATP synthesis/metabolism pathways, as well as actin production or maintenance. The identification of these Dube3a targets is the first step in identifying new pathways regulated by UBE3A in humans that are responsible for the underlying defects in both Angelman syndrome and duplication 15q autism and may reveal new therapeutic targets for the treatment of these disorders.

## Materials and Methods

### *Drosophila* Stocks and Heat Shock

All fly crosses, with the exception of heat shock crosses which were raised at RT, were performed at 25°C on standard corn meal based media. The complete loss of function mutant *Dube3a^15b^* makes no Dube3a protein in the homozygous state and has been previously described [27]. Construction of the UAS-*Dube3a*, UAS-*Dube3a*-C/A, and UAS-*hUBE3A* lines have been described elsewhere [23], [25]. The following stock was obtained from the Bloomington Stock Center (Bloomington, IN): *Heatshock-*GAL4. The progeny of *Heatshock*-GAL4 crosses to various UAS transgene lines were subjected to a 37°C heat shock for 1 hr to induce transgene expression (24-fold increase in Dube3a protein and 37-fold increase in Dube3a-C/A protein) [25]. Flies were allowed to recover for ∼2 min to confirm that they were still alive and then flash frozen in liquid nitrogen for storage at −80°C.

### Proteomic Screen

Heads were removed from frozen flies using molecular sieves under liquid nitrogen as previously described [23]. Cytoplasmic and nuclear proteins were extracted from approximately 300–400 ul of heads using 1X volume of Cytoplasmic Protein Extraction Buffer (CPEB from Bio-Rad). Heads were ground in ice cold buffer to release proteins and then the supernatant (cytoplasmic proteins) collected by centrifugation at 3500RPM at 4°C. The pellet was then re-extracted according to the manufacturers instructions to obtain the nuclear protein extracts. Membrane preparations were made by ultracentrifugation in a sucrose gradient using an established *Drosophila* protocol [28]. For each 50 mg of total protein, 50 ml of 8M Urea plus 8.75 mls of 3–10 pH 40% ampholytes were added to the sample. Samples were prepared for isoelectric focusing in the Rotofor midi-cell according to the manufacturers instructions (Bio-Rad). Liquid phase isoelectric focusing (IEF) was run at a constant 15W until the voltage did not change for three consecutive readings at 15 min intervals. Twenty samples were then simultaneously collected using a vacuum manifold. The pH of each IEF fraction was directly measured using a micro-pH probe and digital pH meter. Urea was then removed from the samples using Vivaspin columns (Sigma-Aldrich). All samples were measured for protein concentration by Bradford assay and then diluted in 1X Laemmli buffer for storage at −20°C.

For each type of extract in each genotype, all protein fractions were visually analyzed across the middle 18 IEF fractions (∼pH 4.5–10.5) by loading ∼2 ug of total protein per lane on a 20 well 4–12% midi-gel in MOPS buffer (Invitrogen). Samples that were similar in pH to within +/−0.1–0.5 pH units were then run side by side on a 4–12% mini-gel for direct comparison of differentially regulated proteins. To quantify fold change in protein intensity the gels were fixed and stained with quantifiable fluorescent protein stain Sypro Ruby (Invitrogen), scanned under UV illumination using a Syngene G-box imaging system and then analyzed using software to measure intensity differences among protein bands (Syngene). Proteins which were determined to change by at least 2 fold+or – were then excised from the gels after Silver staining for visualization on a light box or directly cut from the Sypro gels under UV illumination in some cases.

Peptides were identified from cut bands by matrix assisted laser desorption/ionization time of flight mass spectrometry (MALDI-ToF MS) at either the UTHSC, Tufts University or Harvard Mass Spectrometry facility. Only top hits were considered for inclusion in the list and accuracy was assessed by p-value, Z-score or percent coverage. Protein identifications were performed using various parameters to determine confidence in that particular ID depending on the facility used (i.e Z-score vs pval vs %ID) at the UTHSC Proteomics Core (closed 2007), the Tufts University Proteomics Core (closed 2009) and the Harvard University Proteomics Core.

### Quantitative Western Blot Analysis

Transgenic and control flies, no more than 3 days post eclosion, were subjected to a 37°C heat shock for 1 hr, or no heat shock in the case of *Dube3a^15b^*, and allowed to recover for ∼2 min. and then frozen in liquid nitrogen. Cytoplasmic and nuclear extracts were prepared from fly heads using the ReadyPrep Protein Extraction Kit (Bio-Rad). Samples were resolved on a NuPage 4–12% Bis-Tris 1.5 mm Gel using MOPS Buffer (Invitrogen) and transferred to Immobilon-FL PVDF membrane (Millipore). The membrane was blocked with 5% milk, 3% BSA, and 0.1% Tween-20 in PBS. Primary antibodies were used at 1∶5000: anti-GAPDH (Millipore, cat. #: MAB374), anti-Arginine kinase courtesy of Glen Collier (The University of Tulsa, OK), anti-Eps15 courtesy of Hugo Bellen (Baylor College of Medicine, Houston, TX), and anti-ATPα (cat. #: α5) from the Developmental Studies Hybridoma Bank developed under the auspices of the NICHD and maintained by The University of Iowa, Department of Biology, Iowa City, IA. IR labeled secondary antibodies (α-Guinea pig 680, α-rat 680 and α-mouse 800) were purchased from Li-Cor (Lincoln, NE) and used at a dilution of 1∶10,000. The blot was imaged and analyzed using the Odyssey Infrared Imaging System (LiCor, Lincoln, NE). Samples were normalized using the signal from anti-GAPDH as the reference channel and signal intensity was then adjusted based on the calculated normalization factor assigned to each lane in the channel. Normalized intensities were used for statistical analysis from at least three technical replicates.

### Ubiquitin Enrichment Assays

Ubiquitinated proteins were purified using a polyubiquitin affinity resin (Thermo Scientific) in accordance with the manufacturer’s instructions. In brief, lysates of the appropriate genotype and cellular fraction were incubated with the resin at 4°C overnight on a rotating wheel. The resin was then washed and ubiquitinated proteins were eluted in SDS-PAGE sample buffer for Western blot analysis. Antibody concentrations and conditions were identical to those described above except all secondary antibodies were IR^680^ and no loading control was used since quantification was not possible by this method.

### *In vitro* Ubiquitination Assays

Both Dube3a and ATPα were purified from fly head cytoplasmic extracts using affinity purification protocols. N-terminally FLAG tagged Dube3a was purified using EZview™ Red anti-FLAG M2 Affinity Gel to purify the Dube3a-FLAG fusion protein following manufactures instruction (Bio-Rad). ATPα protein was purified using anti-ATPα monoclonal antibody (Developmental Studies Hybridoma Bank) through immunoprecipitation with immobilized Protein G beads (Pierce) in accordance with the manufacturers instructions. *In vitro* ubiquitination assays were performed as described [29], [30]. In brief, ATPα protein was incubated in a 50 µl reaction (50 mM of Tris-HCl, pH 7.4, 1 mM of DTT, 2 mM of MgCl_2_ and 4 mM of ATP) containing 50 ng of E1 ubiquitin-activating enzyme, 500 ng of E2 ubiquitin-conjugating enzyme (UBCH7), 2 µg of Dube3a-FLAG fusion protein, and 6 µg of bovine ubiquitin. The reaction was incubated at 30°C for 2 h. The reaction was terminated by the addition of SDS-sample buffer, samples were boiled and proteins resolved on a 4–12% Bis-Tris 1.5 mm acrylamide gel using MOPS buffer (Invitrogen) and transferred to an Immobilon-FL PVDF membrane for Western blot analysis. The blots were probed with both anti-Ubiquitin (Millipore) or anti-ATPα (Developmental studies hybridoma bank). Imaging and analysis was performed on an Odyssey Infrared Imaging System (Li-Cor, Linclon, NE). The mean intensity values were measured from the bottom of the ∼100 kDa ATPα band to the top of the gel for three independent experiments. These values were converted to a percentage of the value for LANE 2 (Dube3a+E1/E2 proteins) in order to compensate for any Dube3a auto-ubiquitination signal. The percentages were then imported into Prism version 5.0 for statistical analysis (GraphPad).

### Quantitative Real-time PCR Analysis

Total RNA was extracted from ∼100 ul of fly heads using 200 µl of RNABee solution (AMS biotechnology). RNA was quantified by spectrophotometry (NanoDrop Technologies) and the integrity of the 18S and 26S rRNA verified using an Agilent 2100 Bioanlyzer (Agilent Technologies). Two µg of total RNA were used as input for each cDNA reaction synthesized using random hexamer primers via the High Capacity cDNA Reverse Transcription kit (Applied Biosystems). Intron spanning primers were designed for all 49 potential targets using the Roche Universal Probe Library (http://tinyurl.com/7u6s5bh). Although some of the genes have multiple splice variants, all primers were designed as common assays that would detect all splice-forms, if possible. Three technical replicates were performed for each gene and normalized to the expression of the housekeeping TATA-binding protein (*TBP*) gene. Gene expression was quantified for each cDNA sample in triplicate at 40 ng/reaction using the default cycling parameters of the Roche Light Cycler 480 system. The crossing point (Cp) value for each sample was calculated using Roche absolute quantitation algorithm. The average Cp value among three technical replicates was calculated and the average sample Cp values were normalized for loading by comparison to the average Cp value of TATA-binding protein *TBP*. The fold change (Fc) in gene expression in fly heads was then calculated by comparing the difference of the normalized mean Cp values between *w^1118^* and the *Dube3a^15b^* mutant or between *Heatshock*-GAL4 and each over-expression construct using the equation Fc = (2)^Dcp^.

### Bioinformatic Analysis

Heat maps for visual representation of both protein and transcript fold changes were generated by importing values for fold change for each protein or transcript into the web version of the matrix2png program [31]. Gene Ontology classifications for the list of 43 putative Dube3a regulated proteins was performed using CateGOrizer [32] and more formal pathway enrichment analysis was executed through the Database for Annotation, Visualization and Discovery suite (DAVID) [33]. Functional interaction networks and evolutionary conservation of these proteins was analyzed using the Search Tool for the Retrieval of Interacting Genes (STRING) [34]. Putative transcription factor binding site locations and modules were identified using the Transcription Factor Matrix Explorer web interface [31].

### Muscle Actin Analysis

Wandering 3^rd^ instar larvae of genotypes *w^1118^* or *w^1118^*; *Dube3a^15b^*/*Dube3a^15b^* were dissected in *Drosophila* saline solution, fixed for 20 min in 1X PBS plus 4% paraformaldehyde and 25 mM EGTA followed by one wash in PBS +0.05% Tween-20 (PBST). Fixed larvae were then treated with a 10 min incubation in PBS +0.01% Triton-X 100 (PBSTx) followed by a 20 min incubation in a 500 ul solution containing 3 ul Phalloidin^594^ (Invitrogen) in PBSTx. Larvae were washed in PBST twice and then mounted with Prolong Gold Hardset plus DAPI (Vector Labs) for image capture using a 63X lens under oil immersion on a Zeiss LSM 710 confocal microscope with identical imaging conditions for *w^1118^* and *Dube3a^15b^* genotypes. The tile feature was used to capture the entire A2 muscle region from either the left or right side of the animal (overlap 10%). Z-stacks of 6 µm in depth were used to capture images from the muscle nuclei layer to the top of the muscle surface. A stitched Z-stack section from the middle of this stack was then used for intensity analysis using the Zen image analysis package (Zeiss). Intensity was measured in 150 µm segments perpendicular to the muscle striations in both muscles 6 and 7 from each of four animals per genotype (n = 4). Statistical analysis was performed using a Student’s *t*-test in the Prism 5.0 software package (Graphpad Software).

## Results

### IEF Screen Reveals Fifty Unique Proteins Regulated by Changes in Dube3a or hUBE3A Levels

Using the fly GAL4/UAS expression system [35] we previously made transgenic animals containing human UBE3A splice form II [23] as well as the complete cDNA sequence for fly *Dube3a* and a *Dube3a* mutant that has a single mutation at the active site eliminating the ubiquitin ligase function of the enzyme [25] under the control of the yeast upstream activator sequence (UAS). We also obtained a previously published complete loss of function mutant of Dube3a known as *Dube3a^15b^* which makes transcript, but no protein [27]. Flies under the control of UAS were crossed to a *Heatshock*-GAL4 driver line to induce global high-level gene expression in all tissues. Adult flies no more than 3 days post eclosion were subjected to heat shock to induce transgene expression in the brain and then frozen in liquid nitrogen. In the case of the *Dube3a^15b^* mutant line, flies were grown at 25°C and homozygous *Dube3a^15b^/Dube3a^15b^* flies were used for all experiments. Frozen fly heads were then used to isolate cytoplasmic proteins (soluble fraction), nuclear proteins (re-extracted pellet) and membrane proteins (membrane preparation). **Table 1** summarizes the genotypes, fractions and number of proteins identified during the proteomic screening experiments described in the next section.

**Table 1:** Overview of bands excised, fractions and total number of novel proteins identified.

Fraction		Genotype	Bands Excised	Unique IDs	Unique in Fraction
				per Fraction	
Cytoplasmic		HS-GAL4/+	8		
		HS-GAL4>UAS-*Dube3a*	18		
		HS-GAL4>UAS-*Dube3a*-C/A	18	40	*22*
		HS>hUBE3a-77L	12		
		*Dube3a^15b^*	4		
Nuclear		HS-GAL4/+	2		
		HS-GAL4>UAS-*Dube3a*	6		
		HS-GAL4>UAS-*Dube3a*-C/A	2	12	*5*
		HS>hUBE3a-77L	7		
		*Dube3a^15b^*	10		
Membrane		HS-GAL4/+	27		
		HS-GAL4>UAS-*Dube3a*	3		
		HS-GAL4>UAS-*Dube3a*-C/A	6	24	*6*
		HS>hUBE3a-77L	0		
		*Dube3a^15b^*	14		
Found in Multiple Fractions					*17*
			137	76	50

**Figure 1:**
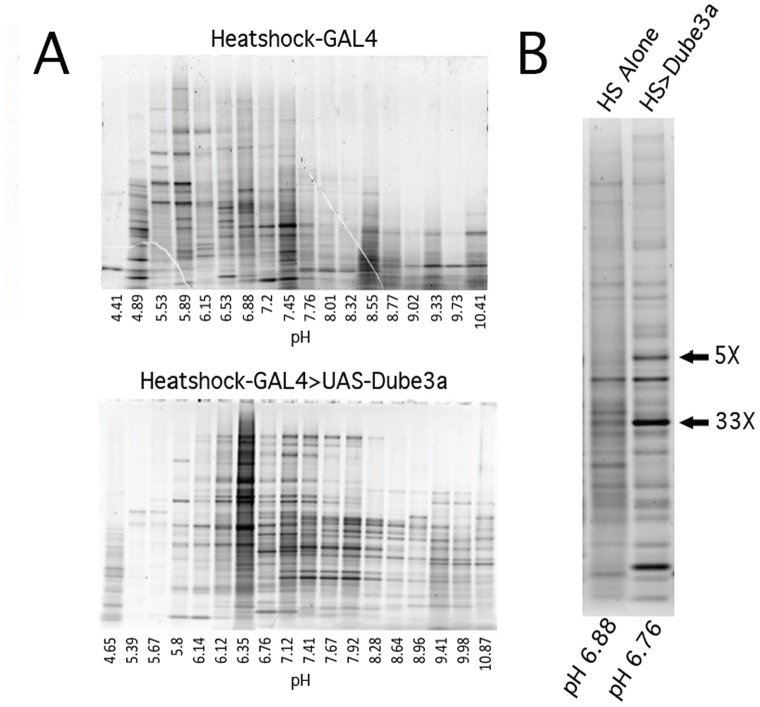
Identification and quantification of fold change in potential Dube3a targets. **A)** The middle 18 (out of 20) fractions from the first dimension liquid isoelectric focusing cells were run on 4–7% polyacrylaminde gels to separate proteins in each pH fraction by molecular weight and to determine what adjustments would be needed to normalize protein bands across fractions. Direct comparisons between matched pH samples were made between either wild type and *Dube3a^15b^* loss of function extracts or *Heatshock*-GAL4 and *Heatshock-*GAL4>UAS-*Dube3a*, *Dube3a*-C/A or *hUBE3A* extracts. This figure illustrates proteins separated by isoelectric point (pI) and their measured average pH for each fraction from *Heatshock*-GAL4 alone on the top and *Heatshock*-GAL4>UAS-*Dube3a* on the bottom. Fractions that were approximately pH matched were then run on separate PAGE gels for direct comparisons. **B**) Direct comparison of *Heatshock*-GAL4 Alone pH 6.88 to *Heatshock*-GAL4>UAS-*Dube3a* pH 6.76 cytoplasmic protein extracts. Note the bands with the arrows indicating proteins that went up by 5-fold (∼51 kDa band) or 33-fold (∼37-kDa band) in the *Dube3a* over-expressing extracts. These bands, and others like it, were excised from the polyacrylamide gels and identified by MALDI-ToF ToF analysis. These particular proteins were identified as the predicted 53 kDa form of CG7430 and the predicted 39 kDa form of Arginine kinase (CG32031).

Proteins from each genotype were extracted as described in the methods section. Proteins from each fraction were then separated by isoelectric point using a liquid phase isoelectric focusing cell (IEF). The actual pH and protein concentration for all twenty fractions from IEF cell were recorded and then each fraction run in a second dimension separation by molecular weight. **Figure 1A** illustrates a second dimension PAGE separation (∼pH 4.5–pH 10.5) for the middle eighteen IEF fractions in *Heatshock*-GAL4 alone (top) and *Heatshock*-GAL4>UAS-*Dube3a* cytoplasmic fraction proteins (**Figure 1A**). Protein fractions were then approximately matched by pH (within at least +/−0.5 pH units) in control versus over-expression and control versus *Dube3a^15b^/Dube3a^15b^* heads and then run next to each other to separate proteins in that fraction by molecular weight for the quantification of intensity changes. We compared proteins in each fraction using the quantitative fluorescent protein stain Sypro Ruby and imaged these gels for comparisons (**Figure 1B**). Protein bands that changed intensity between control and experimental in either direction by at least 2-fold were then excised from the gels for identification by mass spectrometry. The proteins illustrated in **Figure 1B** were approximately 51 kDa and 38 kDa. They increased by 5-fold and 33-fold as compared to *Heatshock*-GAL4 alone in a *w^1118^* background. These protein bands were cut from the *Dube3a* over-expression lane and identified as uncharacterized protein CG7430 and the Arginine kinase protein (ArgK: CG32031). Using this approach we identified 137 bands to cut from these gels comprising some 76 individual proteins identified by mass spectrometry (**Table 1**). In all, using this screening method we found 22 proteins unique to the cytoplasmic fractions, 5 unique to the nuclear fractions, 6 unique to the membrane fractions and 17 proteins that were identified in multiple fractions. Grouping all fractions together revealed 49 unique proteins that changed in expression level under conditions of increased or decreased Dube3a levels or human UBE3A levels in the brain. We extracted functional information on these hits from Flybase (www.flybase.org) in order to compile a comprehensive list of protein identifications, the cellular fraction in which they were identified, pH, relative fold change, apparent molecular weight as well as database identifiers (see **Table S1** for complete details).

**Figure 2:**
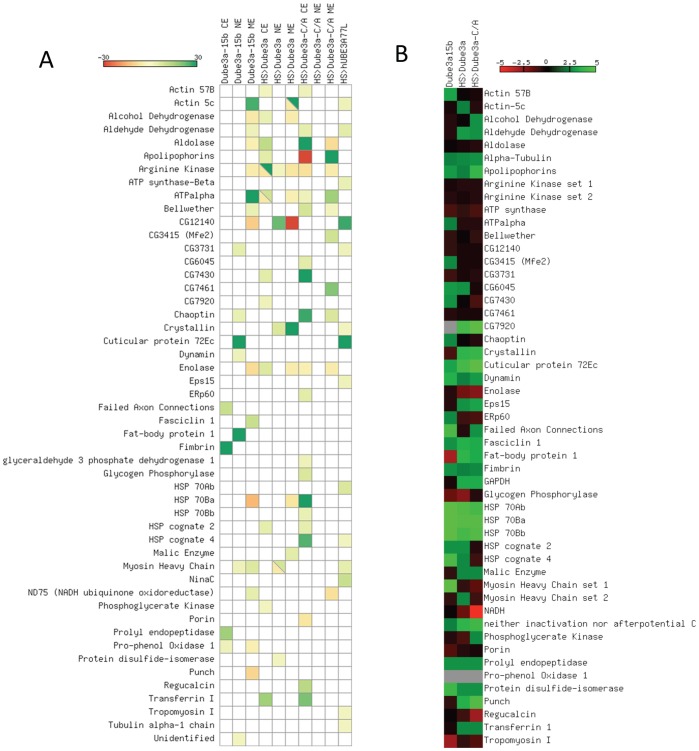
Heatmap visualization of protein and transcriptional Dube3a dependent changes. **A)** Fold change for proteins identified as in Figure 1 was calculated using fluorescent intensity as compared to *Heatshock*-GAL4 alone or w- extracts. The scale is from 30-fold decrease (red) to 30-fold increase (green) in intensity with yellow as an intermediate on the scale. Boxes represent individual proteins identified by mass spectrometry for that fraction (CE, cytoplasmic; NE, nuclear; and ME, membrane) and in that particular genotype. If a particular protein was identified several times in that fraction at different molecular weights or pH values, the average of these intensities was used for the purposes of the heatmap (for complete details on each protein see **Table S1**). In three cases, a particular protein was determined to go up in one experiment, but down in another for a different isoform. These proteins are indicated with a slash through the box and the two opposing colors filled into each half. **B)** Average fold change by qRT-PCR for genes identified in the screen under varying levels of Dube3a protein expression. Although some of these genes have several predicted spliceforms, only common assays that could identify as many transcripts as possible were used for this analysis. In the case of Arginine kinase primer set 1 recognizes ArgK-RC and RF while set 2 recognizes RA and RB. For Myosin Heavy Chain set 1 includes RA-C, RF-RL and set 2 RB, RD-E, RK, RM and RJ. The scale is from 5-fold or greater decrease (red) to 5-fold or greater increase (green) in transcript vs. control (either *Heatshock*-GAL4 alone or *w^1118^*). Note that some genes showed transcriptional changes greater than 5-fold, so they are on the upper or lower end of the color scale. A complete table of qRT-PCR results can be found in **Table S2**, including error bars for the triplicate technical replicates.

Many of the proteins identified in our screen changed intensity in multiple cellular fractions or under both elevated and decreased levels of *Dube3a*. In order to visualize the effects of changes in *Dube3a* expression on individual proteins identified we constructed a heat map for the fold change of each protein under each condition of Dube3a over/under expression and in cytoplasmic, nuclear and membrane fractions (**Figure 2A**). According to current dogma the over-expression of wild type Dube3a should result in decreased levels for proteins that are direct ubiquitin targets degraded by the ubiquitin proteasome and, therefore, we expected that most of the proteins identified in the screen would decrease intensity as compared to the *Heatshock*-GAL4 control extracts. We found instead that most of the proteins identified in our screen increased intensity as a result of both decreased and increased levels of Dube3a (**Figure 2A**). A notable exception is the 54-fold decrease in protein CG12140 in membrane fraction but 24-fold increase of this protein in nuclear fraction upon wild type Dube3a over-expression. Similarly, the protein Apolipophorins (CG11064) increased intensity by 33-fold in cytoplasmic fraction and decreased 390-fold in membrane fraction when the *Dube3a*-C/A mutant was expressed in fly heads. Both of these examples may represent a shift in protein localization from one cellular compartment to another in a Dube3a regulated manner.

Six proteins increased by >20 fold in various cellular fractions from the *Dube3a^15b^* mutant: Actin5c (CG4027), ATPα (CG5670), Cuticular protein 72Ec (CG4784), Fat-body protein (CG17285), Fimbrin (CG8649), and Prolyl endopeptidase (CG5355). Elevated protein expression in the mutant may indicate that these particular targets are de-repressed by loss of Dube3a, and thus could be direct ubiquitin targets degraded by the ubiquitin proteasome system. The most likely direct ubiquitin targets on this list are ATPα and ArgK since these proteins have previously been identified as a proteins ubiquitinated in the developing fly nervous system [36].

Six proteins only showed differential protein expression in the presence of elevated human UBE3A, which was used previously to identify both *Punch* and *Pebble* targets [23], [25]. These proteins include the endocytic recycling protein Eps15 (CG16932), heatshock protein HSP70Ab (CG18743), ATP synthesis protein ATP synthase-α (CG11154), structural cellular components Tubulin α-1 chαin (CG1913) and Tropomyosin I (CG4898) as well as the myosin-like eye specific protein kinase protein NinaC (CG5125). All of these proteins except Eps15 and NinaC are directly related to structurally similar proteins identified by changes in Dube3a levels such as HSP70Ba (CG31449), ATP synthase-α (CG3612) and myosin heavy chain (CG17927). Thus evolutionary differences between fly and human UBE3A resulted in the identification of similar types of proteins structurally, but the human UBE3A protein was not able to target the appropriate form of these proteins in some cases.

### Quantitative Real-time PCR Reveals Eight Potential Transcriptional Targets of Dube3a

Gene expression was analyzed for all 49 proteins identified during the proteomic screen using primer sets common to all splice forms of these genes. Seventeen genes did not demonstrate appreciable changes in gene expression (≥1.5-fold up or down) under any conditions of varying Dube3a protein levels and one gene, Pro-phenol Oxidase 1, could not be analyzed using several different primer sets (**Table S2**). A heat map analysis of all of the transcripts is illustrated in **Figure 2B** **.** Thirteen genes showed a >1.5-fold increase in gene expression when the Dube3a-C/A protein was expressed, indicating that the ubiquitin ligase function of Dube3a is not required for increased expression of these particular proteins. Eleven of these genes also responded in a positive fashion to increased wild type Dube3a levels. Eight of these genes responded in a positive direction to increased expression of *Dube3a*-C/A and a negative manner to loss of Dube3a (i.e. *Dube3a^15b^*), or not at all, including the GTP cyclohydrolase I gene which we previously described [25]. This group of genes, with the exception perhaps of the three Hsp70 genes that responded by >25 fold increase to loss of Dube3a, are potentially regulated by Dube3a through the relatively uncharacterized transcriptional co-activation function of the Dube3a protein in a ubiquitin ligase independent manner.

Three Heatshock protein encoding genes (*Hsp70Ab, Hsp70Bb* and *Hsp70Ba*) showed a >25-fold increase in gene expression in the *Dube3a^15b^* mutants (**Figure 2B** **and Table S2**), while a much smaller 2.6-fold increase in gene expression was observed for the heatshock related gene *Heatshock Cognate 4*. Since neither the *w^1118^* line or the *Dube3a^15b^* line were subjected to heatshock, these results indicate that heatshock proteins may be indirectly regulated by Dube3a through a transcription factor intermediate or that Dube3a itself can confer transcriptional co-repression.

We next searched for the presence of transcription factor binding sites in the eight genes that showed increased transcription when we expressed Dube3a-C/A but either were not affected by or showed decreased expression in *Dube3a^15b^* mutants. These genes have the most potential for binding transcription factors that could interact with Dube3a to cause transcriptional co-activation. Using the Transcription Factor Matrix Explorer (TFM-Explorer) we identified significant enrichments (pval≤0.005) for transcription factor modules shared by these eight genes: *Apolipophorins, Crystallin, Eps15, Fasciclin 1, Fat-body protein 1, glyceraldehyde-3-phosphate dehydrogenase, neither inactivation nor afterpotential C* and *Pu* [37]. These eight genes are marked with an asterisk in **Table S2**. TFM-Explorer analysis revealed 17 clusters with a significant enrichment for 12 different transcription factor binding sites in the promoter sequences of these eight genes (**Figure S1**). The top five hits included binding sites for transcription factors glial cells missing (GCM: pval = 1.1×10^−4^), Chorion Factor II (CF2: pval = 2.6×10^−4^), Heatshock Factor (HSF: pval = 8.3×10^−4^), Abdominal B (Abd-B: pval = 1.4×10^−3^) and Ultrabiothorax (Ubx: pval = 1.5×10^−3^).

The glial cells missing transcription factor is both necessary and sufficient for the production of glial cells versus neurons in both the central complex of the adult *Drosophila* brain and the developing embryo [38], [39]. The HSF transcription factor binding sites are located in genes that undergo rapid response to stimulus, environment or require orchestrated expression during a biological time-point or process such as may occur during synaptic growth or retraction, while the CF2 transcription factor is closely associated with actin development in larvae [40]. This analysis suggests that Dube3a may regulate actin cytoskeleton and possibly even neuronal regulatory pathways though co-activation of genes under the control of one or more of these transcription factors.

### Proteins Identified are Involved in Nervous System Development, Actin Cytoskeleton Remodeling, ATP Synthesis/Metabolism and Carbohydrate Synthesis/Metabolism

**Figure 3:**
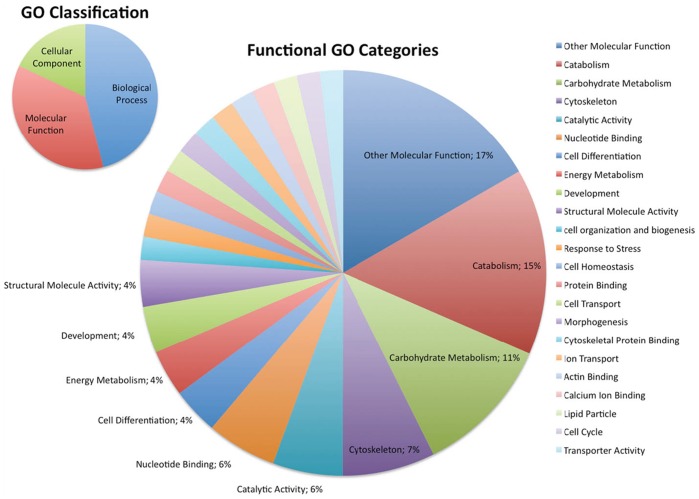
Gene Ontology (GO) characterization of potentially Dube3a regulated proteins. The GO classifications for each of the 43 proteins affected by changes in Dube3a expression or the expression of the ubiquitin defective Dube3a-C/A construct were extracted from Flybase (www.flybase.org) and then input as a list into CateGOrizer (http://tinyurl.com/79kzqn9) for counting of the classification groups. The small pie chart in the upper left corner indicates the root GO categories represented in our data (18% cellular components, 46% involved in a biological process, and 36% involved in a particular molecular function). The larger pie chart illustrates the major categories of molecular function found in this data set. Most notable are the Catabolism, Carbohydrate Metabolism, Cytoskeleton, Nucleotide Binding, Energy Metabolism and Development categories which comprise ∼47% of the proteins identified in the screen. The colored slices with no percentage listed are all less than 2% of the total set of proteins. All of the categories are listed with a color-coded legend to the right of the pie chart.

We classified all 49 proteins from the screen using the specific Gene Ontology (GO) classification term culled from Flybase for each hit. A general root classification of the proteins we found indicates that 18% of the proteins from the screen are cellular components, 36% are involved in a molecular function and 46% are part of a specific biological process (**Figure 3**, **inset**). Finite analysis of the GO terms for each protein indicated that the major cellular functions regulated by these proteins include carbohydrate metabolism (11%) as well as energy metabolism (15%), actin cytoskeletal structure (7%), development (4%), and nucleotide binding (6%) (**Figure 3**). Some proteins were also classified in related processes to these key groups such as actin binding (2%), cytoskeletal protein binding (2%), cell transport (2%), morphogenesis (2%), and biogenesis (2%).

Several proteins on the list of regulated candidates were immediately of interest, as they are specifically known to be involved in neuronal development and activity. These proteins include the dopamine regulatory protein Punch, which we previously showed to be regulated by Dube3a *in vivo* [25], the axon guidance proteins Fasciclin 1 (CG6588) and Failed Axon Connections (CG4609), the endocytic recycling proteins Dynamin (CG18102) and Eps15 (CG16932), the creatine kinase orthologue Arginine kinase (CG032031) and the axon homeostatic regulator Na+/K+ pump ATPα (CG5670).

The next most interesting groups of proteins were Heatshock and Heatshock cognate proteins. The identification of changes in Heatshock proteins is not an artifact of the *Heatshock*-GAL4 induction in these cases since all flies containing *Heatshock*-GAL4 were subjected to the same heatshock protocol and the control flies contained the *Heatshock*-GAL4 driver for comparisons of band intensities. In addition, we identified at least one Heatshock protein (Hsp70Ba (CG31449)) that decreased in *Dube3a^15b^* mutants as compared to wild type outside the context of the heatshock protocol. We also found on our list 4 proteins that make up the actin cytoskeleton or muscle including Actin5c (CG4027), Actin57B (CG10067), and myosin heavy chain (CG17927) as well as Tropomyosin I (CG4898). Finally, there were 11 proteins involved in more basic metabolic processes such as dehydrogenases, isomerases and kinases. Since the over-expression of human UBE3A only resulted in changes to 6 proteins that were not also regulated by changes in Dube3a, we conducted more in depth functional analysis using just the 43 proteins in the Dube3a regulated set.

**Figure 4:**
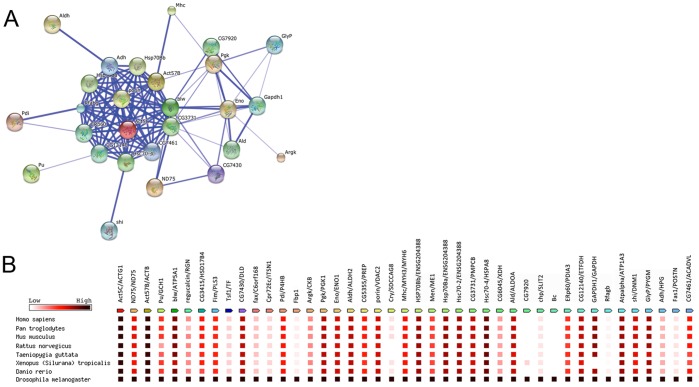
String and phylogenetic analysis of putative Dube3a regulated proteins reveals a tight cluster of proteins involved in stress response, actin cytoskeleton and ATP binding. **A)** All 43 proteins were submitted for STRING analysis and 27 of these proteins formed a single cluster of proteins related by function, pathway or literature search. The thickness of the blue line represents the predicted strength of the relationship between any two proteins. The tight cluster at the center of the diagram consists of 13 proteins enriched for involvement in stress response (Bonferroni corrected pval = 8.01E−03), molecular chaperones (Bonferroni corrected pval = 1.64E−05), ATP binding (Bonferroni corrected pval = 1.11E−04) and mitochondrial membrane proteins (Bonferroni corrected pval = 4.98E−01). **B)** Analysis of protein homology among common model organisms and humans for the Dube3a regulated protein set of 43 proteins from the proteomic screen. The gene name on the left is the fly gene and on the right of the (/) is the human gene name. The scale is from low homology (white) to high homology (black) with red being ∼50% protein sequence identity. Over half of the proteins (67%) were conserved by at least 50% identity from fly to human homologues. Many of these proteins were also conserved in other model organisms, indicating that these potential UBE3A targets could be studied in other systems to validate the results of our proteomic screen in flies. At least seven of the proteins (16%) showed almost no homology (less than 10%) to proteins in other non-insect organisms and are therefore of considerably less interest in terms of UBE3A involvement in human disease mechanisms.

A more formal analysis of these 43 proteins identified from the screen was done using pathway enrichment analysis tools within the Database for Annotation, Visualization and Discovery suite (DAVID) [33]. Using the “medium” stringency setting we analyzed the putative Dube3a regulated proteins as a group. Eight clusters of categories were identified from the set of potential Dube3a target proteins with enrichment scores (Es) ≥1.0 and uncorrected pval ≤0.05 (**Table S3**). These included an enrichment for nucleotide binding proteins (Es = 4.1), Heatshock proteins (Es = 3.8), glycolysis (Es = 3.7), oxidation/reduction (Es = 3.3), actin cytoskeleton (Es = 2.9), homeostasis (Es = 2.1), actin binding (Es = 1.8) and mitochondrial oxidative phosphorylation (Es = 1.6). This same set of 43 proteins was then used as an input list for STRING analysis (http://string.embl.de/) in order to identify relationships among proteins that may reveal protein networks regulated by Dube3a. Confidence relationships among these proteins revealed a tight cluster of 13 proteins, some of which are involved in actin cytoskeleton remodeling and some in cellular trafficking, as well as a wider network comprising 27 proteins including the neurotransmitter regulator Punch (CG9441) and the axonal transport protein shibire (dynamin: CG18102) that are interrelated by direct physical interaction or functional association (**Figure 4A**). The cluster on the right side of the figure contains mostly metabolic proteins involved in ATP synthesis and catabolism with a central node at the enolase (CG17654) protein. Finally, we performed phylogenic analysis on this list of 43 proteins to determine which of these targets may also be targets of human UBE3A (**Figure 4B**). We found that 62% of these fly proteins shared >50% sequence identity to their human homologues. Six of these are novel proteins previously uncharacterized in *Drosophila* but clearly homologous to human proteins. As expected, some proteins such as Crystallin (CG16963), Pro-phenol Oxidase 1 (Bc: CG42639), Cuticular protein 72Ec (CG4784) and apolipophorins (Rfabg: CG11064) are involved in fly specific structures and functions and thus do not have any appreciable homology to humans and other model organisms. Our gene ontology and phylogenetic analysis indicates that Dube3a may be a key regulator of multiple proteins that regulate cellular trafficking through actin cytoskeletal proteins requiring ATP synthesis and metabolism to function in both flies and humans.

### ATPα is Regulated by the Ubiquitin Ligase Function of Dube3a

**Figure 5:**
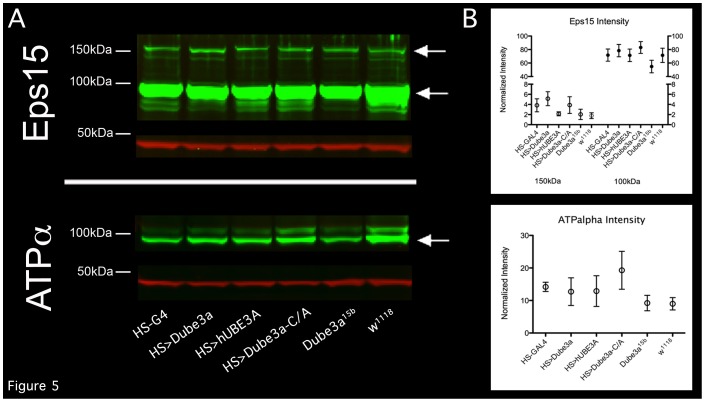
Quantitative Western blot analysis of potential Dube3a targets Eps15 and ATPα. **A)** IR Western blots of Eps15 and ATPα (green) using a anti-GAPDH loading control (red). Quantification of the normalized band intensities for the known protein isoforms of Eps15 and ATPα are graphed in **B**. Genotypes for extracts in each lane are labeled at the bottom of the blot and graphs. White arrows point to the Eps15 ∼150 kDa and ∼100 kDa bands (top) as well as the ∼100 kDa ATPα band. Note a slight increase in intensity for the ∼150 kDa Eps15 band in the HS>Dube3a lane and slight decrease in the ∼100 kDa Eps15 band in *Dube3a^15b^* mutants as compare to *w^1118^*. Also, a decrease in ATPα can be seen on this particular blot in *Dube3a^15b^* mutants as compare to *w^1118^.* **B**) Graphs of the normalized fluorescent intensity values for each band. Note a trend towards decreased ∼150 kDa Eps15 in the hUBE3A overexpression lane and decreased ∼100 kDa Eps15 in the *Dube3a^15b^* mutant as compared to *w^1118^*. These results did not reach significance in triplicate replicates.

In order to investigate direct effects of Dube3a ubiquitin ligase function on potential protein substrates identified in our screen we looked for changes in protein expression or ubiquitination state under differential Dube3a expression conditions. Although antibodies were available for Eps15, Arginine kinase, and ATPα for Western blot analysis, only the Eps15 and ATPα antibodies revealed appreciable protein stability/expression changes that were Dube3a dependent (**Figure 5A**). Appreciable differences could be observed by Western blot for both the ∼150 kDa and ∼100 kDa forms of the Eps15 protein (**Figure 5A** **, top**). Changes in the expression of Dube3a did cause fluxuations in the normalized intensity levels for ATPα, but these changes were variable among the four independent technical replicates and did not reach significance (**Figure 5A** **, bottom and 5B, bottom**).

**Figure 6:**
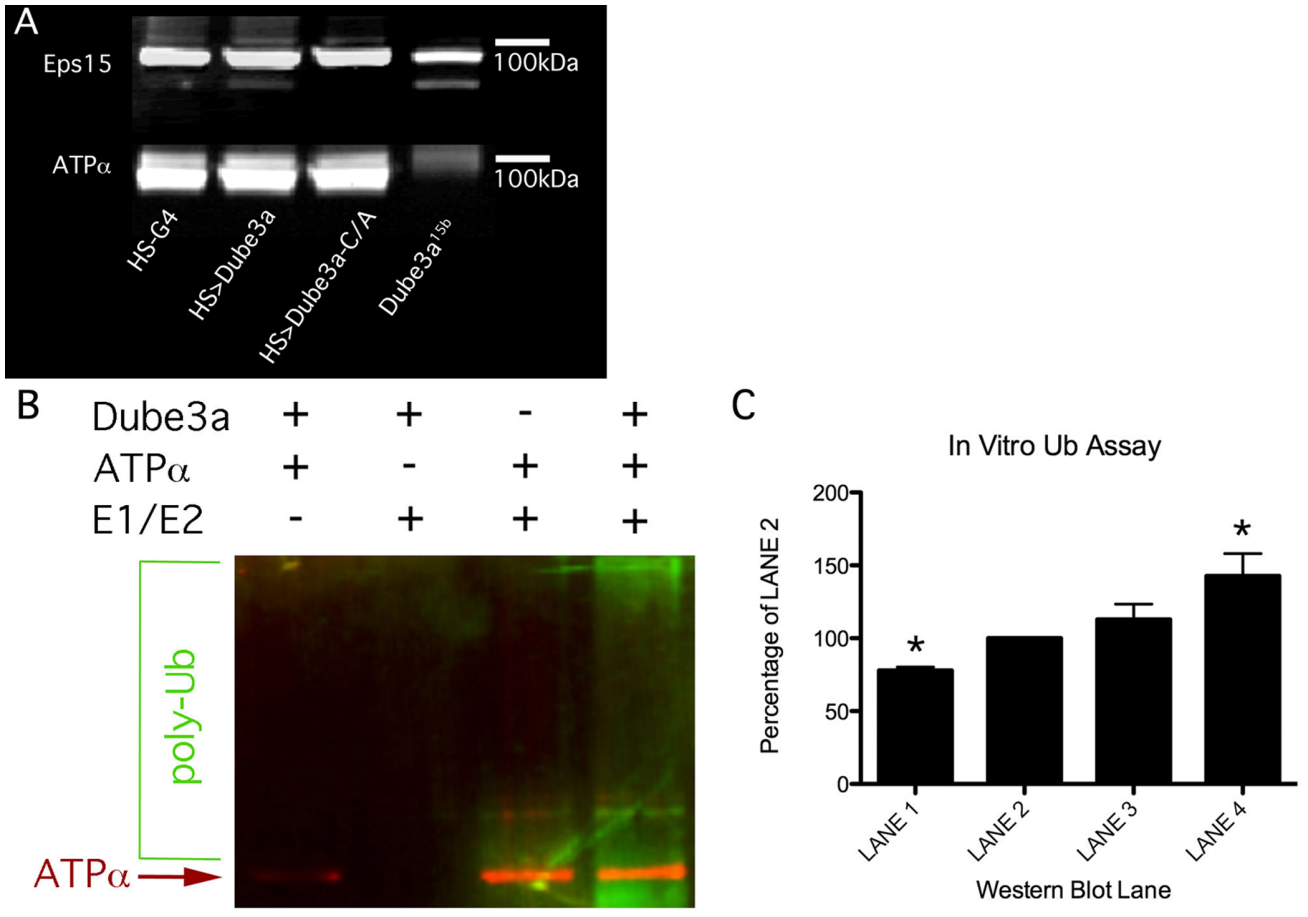
Ubquitination state of Eps15 and ATPα. **A)** Ubiquitin enrichment from cytoplasmic extracts with elevated or decreased levels of Dube3a. Genotypes are listed below. Western blots were performed on ubiquitin-enriched extracts using Eps15 (top) and ATPα antibodies (bottom). Only the ∼100 kDa form of Eps15 could be detected in the elutions from the ubiquitin enrichment column for all genotypes. No ∼150 kDa form could be detected. The ∼100 kDa form appears to be ubiquitinated even in the *Dube3a^15b^* mutant fractions. Ubiquitinated ATPα could be detected in all fractions except for the *Dube3a^15b^* lane, indicating that ATPα may be ubiquitinated in a Dube3a dependent manner. **B)** *In vitro* ubiquitin assays. Purified Dube3a and ATPα proteins were mixed with E1 and E2 proteins *in vitro* in an attempt to ubiquitinate ATPα using the Dube3a E3 ubiquitin ligase. The (+) and (–) symbols above the Western blot indicate the presence or absence of a component in the ubiquitination reaction run in that lane. ATPα protein could be detected in all of the appropriate lanes (red) and a Poly-Ub smear could be seen in the fourth lane from the left (the reaction which contained all components). **C)** The green signal (poly-Ub antibody) was used to quantify the level of ubiquitination detected above background (LANE 1) and above any Dube3a auto-ubiquitination (LANE 2). A strong smear detected in LANE 4 was 143%+/−15% above the background levels and was significantly higher than any signal that comes from Dube3a auto-ubiquitination (LANE 2). The (*) symbols indicates a p-value of less than 0.05 for these results by one way ANOVA analysis.

To determine if the Eps15 and ATPα proteins are ubiquitinated *in vivo* we used a ubiquitin enrichment column to pull down all ubiquitinated proteins in cytoplasmic protein fractions expressing wild type Dube3a, Dube3a-C/A or in a complete loss of function *Dube3a^15b^* background (**Figure 6A**). We found that elevating Dube3a expression did not appreciably change the amount of ubiquitinated Eps15 or ATPα detected in the elution from the ubiquitin enrichment column, but that complete loss of Dube3a (in *Dube3a^15b^* homozygotes) resulted in a decrease in the amount of ubiquitinated ATPα detectable in the elution as compared to wild type (*w^1118^*;HS-GAL4) head extracts (**Figure 6A**). No significant changes in the ∼100 kDa form of Eps15 were detected and the ∼150 kDa form, which showed variable expression by Western blot, was not detectable in the eluate of the ubiquitin enrichment column. These results suggest that ATPα is ubiquitinated in a Dube3a dependent manner, but Eps15 is not.

To determine if ATPα can act as a direct substrate for the Dube3a E3 ubiquitin ligase we performed *in vitro* ubiquitin assays using Dube3a and ATPα proteins purified from fly head cytoplasmic extracts combined with recombinant E1 ubiquitin activating enzyme and E2 ubiquitin-conjugating enzyme [29], [30]. The addition of purified Dube3a and ATPα in the absence of E1/E2 enzymes did not produce a ubiquitin smear as detected using a anti-Ubiquitin polyclonal antibody (**Figure 6B** **, LANE 1**). The addition of purified Dube3a plus E1/E2 proteins did produce a Ub signal slightly above background (**Figure 6B** **, LANE 2**). The signal in LANE 2 was considered the detection threshold above background (100%) since recombinant UBE3A is known to trans-ubiquitinate itself *in vitro* [41]. A faint poly-Ub smear could be detected when purified ATPα was combined with E1/E2 proteins (**Figure 6B** **, LANE 3**) and a strong smear appeared with the addition of Dube3a protein (**Figure 6B** **, LANE 4**). This experiment was repeated three times using three different purifications. One Way ANOVA analysis with Tukey’s multiple comparison testing among the four groups indicated that there is a significant difference among the groups (pval = 0.0077) and that significantly less poly-Ub is detected in the lane containing no E1/E2 proteins (**Figure 6C** **, 78+/−2%, pval ≤0.05, LANE 1**) while significantly more poly-Ub is detected in the lane with all three components (**Figure 6C** **, 143+/−15%, pval ≤0.05, Lane 4**) as compared to the control lane which lacks the ATPα substrate (**Figure 6B** **, 100%, LANE 2**). These results indicate that ATPα may act as a direct substrate for ubiquitination by the E3 ubquitin ligase Dube3a *in vitro*.

### Dube3a is Involved in Muscle Actin Structural Integrity in Fly Larvae

**Figure 7:**
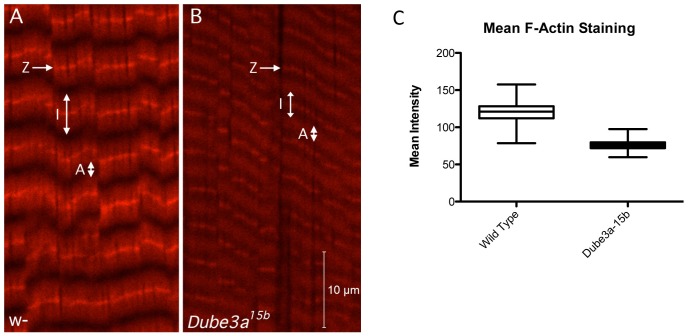
Actin deficiency in the body wall muscles of *Dube3a^15b^* larvae. **A)** Confocal maximum projection image from wild type (*w^1118^*) 3^rd^ instar larval body wall muscle stained with Phalloidin^594^ to visualize filamentous actin. Letters and arrows indicate the Z-line, I-band and A-band of the muscle sarcomere. Fluorescent staining intensity was measured across a 150 mm span in both *w^1118^* (**A**) and *Dube3a^15b^* mutant larvae (**B**). Note the change in fluorescent intensity as well as a shortening of the I-band and a loss of A-band resolution in the mutant (**B**) as compared to wild type (**A**). **C**) Quantification of filamentous actin staining for n = 4 animals per genotype. Fluorescent intensity was measured across 150 µm perpendicular to the Z-bands in four *w^1118^* and four *Dube3a^15b^* larva. The mean fluorescent intensity for Ph^594^ was 37% less in the *Dube3a^15b^* mutant animals (*t*-test, pval ≤0.001).

Since both bioinformatic and qRT-PCR analysis implicated a role for Dube3a in the regulation of the actin cytoskeleton genes *actin5c*, *actin57B*, *Mhc*, and *Tm1* we investigated the effects of decreased Dube3a levels on larval muscle. Crawling 3^rd^ instar larvae have a large area of easily accessible body wall muscle that can be visualized with the filamentous actin (F-actin) toxin phalloidin. We dissected and fixed four wild type and four *Dube3a^15b^* mutant larvae and stained the body muscles with fluorescently labeled phalloidin (Ph^594^). The mean fluorescent intensity was 37% lower in the *Dube3a^15b^* mutants (pval≤0.0001) than in wild type animals and the range of signal intensity was lower as well (**Figure 7**). While the Z-line staining intensity clearly was lower in the *Dube3a^15b^* mutants, we also observed a merging of adjacent I-bands making it difficult to clearly identify A-bands in these animals (**Figure 7**). These results indicate that Dube3a is involved in either the production or stability of muscle actin filaments in flies in addition to a probable role in neuronal actin cytoskeletal components.

## Discussion

Changes in *UBE3A* gene and protein expression levels in neurons are responsible for the primary underlying molecular defects in AS and 15 q duplication autism in both humans [7], [42] and mouse models [11], [19]. Although it has been clear for some time that it is the underlying network of proteins or genes regulated by UBE3A that are responsible for the neurological phenotypes observed in these two conditions, no comprehensive screen for UBE3A regulated targets has been conducted until now. Here we have demonstrated the utility of *Drosophila* to identify 43 potential Dube3a targets in the nervous system that may be relevant to the human conditions. It should also be noted that 52% (26/50) of the unique proteins identified by our screen did not change in any cellular fraction in the *Dube3a^15b^* mutant and of those that did, only 12 out of 24 proteins (50%) changed intensity by >10-fold validating our prediction that the proteomic analysis of complete loss of function mutants alone would not have revealed the majority of proteins that are actually regulated by Dube3a in a temporal specific manner. More importantly, we have now uncovered sets of proteins involved in the process of actin cytoskeletal dynamics as well as ATP driven metabolic and catabolic pathways which will stimulate new avenues of research in existing fly and mouse models of AS and autism.

Although we found that both actin and myosin transcripts were elevated in *Dube3a^15b^* loss of function animals, we also found that Tropomyosin I (Tm1) levels decreased by 3.3-fold in the absence of Dube3a protein (**Table S2**). This may be the cause of the decrease in filamentous actin we found in larvae since Tm1 physically interacts with and stabilizes actin filaments [43]. The identification of actin cytoskeletal components regulated by changes in Dube3a levels is significant on several levels. First, in the original study describing the *Dube3a* loss of function mutations in flies the authors found that adult flies showed locomotor defects, specifically climbing ability [27]. They attributed these defects primarily to the accumulation of Dube3a substrates in the nervous system. We found, however, that muscle integrity is compromised in *Dube3a* deficient larvae and most likely in adult muscle as well, suggesting that muscle defects may also contribute to the locomotor defects observed in these animals. Second, the upstream regulation by *Dube3a* of actin cytoskeleton through fly Pebble or UBE3A regulation of ECT2 may explain the changes in actin cytoskeletal components we found in our screen since Pbl/ECT2 is a Rho-GEF which is a master regulator of the cytoskeleton. Although we did not identify Pbl by liquid phase isoelectric focusing in the current screen, we previously showed that Pbl/ECT2 physically interact with both fly and human Dube3a/UBE3A proteins [23]. Third, mice deficient for *Ube3a* protein have significant defects in experience dependent synaptic plasticity in the visual cortex [15], suggesting a connection between loss of Ube3a and the inability to regulate the actin cytoskeleton appropriately during synaptogenesis. Finally, although some variability has been observed for dendritic spine density in *Ube3a* deficient animals [20], [22] several studies indicate that loss of *Ube3a* can result in a decrease in dendritic spine density on basal pyramidal neurons [15], [18], [20]. Our findings that Dube3a can affect appropriate levels of actin and myosin in the fly brain in addition to the defects in muscle integrity we observed in Dube3a deficient larvae supports the hypothesis that UBE3A is an upstream regulator of the actin cytoskeleton which can cause synaptic defects when both under expressed (as in AS) or over-expressed (as in int dup(15) syndrome).

Our current screen for Dube3a regulated proteins in the fly nervous system suggests a role for Dube3a in the regulation of actin filaments through the regulation of actin, beta-tubulin and heat shock proteins. In a previous screen for ubiquitinated proteins in the developing fly nervous system several actin filament regulatory proteins were identified. These included not only heat shock proteins Hsp83, Hsp27 and Hsp26 as well as alpha-tubulins 84D and 67C but also several nervous system specific regulators of axon guidance such as neuroactin, fasciclin (2 and 3), and failed axon connections [36]. In fact, given the limits of any proteomic screen in distinguishing almost identical proteins from a large family like the heat shock and tubulin proteins it is possible that Dube3a actually targets the same heat shock and tubulin proteins which were identified in the previous screen. It is encouraging that at least some of the same proteins identified in this Dube3a specific screen were also identified as proteins directly ubiquitinated in the fly nervous system by Franco *et al.* (**Table 2**). Additional experiments performed in this study indicate that more ubiquitinated ATPα is observed in the presence of functional Dube3a in an *in vitro* ubiquitin assay making it the first valid ubiquitin target of Dube3a in the fly nervous system. However, we must point out that additional experiments similar to those performed by Franco *et al*. *in vivo* will be required to establish direct ubiquitination of ATPα by Dube3a in neurons.

**Table 2:** Dube3a regulated proteins identified in this study that are ubiquitinated in the nervous system according to Franco *et al.* 2011 [36].

Dube3a Screen	Bio-Ub Screen
Arginine kinase	Arginine kinase
ATPalpha	ATPalpha
beta-Tubulin	alpha-Tubulin (84C and 67C)
HSP70 (Ba and Bb)	HSP70 Cb
*Fascillin 1*	*Fascillin (2 and 3)*
Faild Axon Connections	Faild Axon Connections
HSP cognate-4	HSP cognate-4
Enolase	Enolase

In this study we show a >25 fold increase in gene expression for Hsp70Ab, Hsp70Ba and Hsp70Bb in a homozygous *Dube3a^15b^* loss of function mutant (**Table S2**). A link between the ubiquitin proteasome system and the heat shock response has been known for some time [44], [45]. In fact, transcriptional regulation of Hsp70 may be regulated by another E3 ubiquitin ligase called CHIP (the C terminus of Hsp70-interacting protein) which can physically interact with the transcription factor HSF1, a primary regulator of heat shock protein transcription [46]. A direct physical interaction between UBE3A and Hsp70 has also been established and is thought to provide a connection between Hsp70 bound mis-folded proteins and UBE3A ubiquitination of these substrates destined for degradation by the UPS [47]. This induction of Hsp70 gene transcription in the absence of Dube3a protein may be a regulatory response driving the use of other, possibly less specific, E3 ligases such like CHIP for the clearing of mis-folded proteins in the absence of Dube3a [48].

Perhaps the most significant single target identified in this study regulated by Dube3a is the Na+/K+ pump ATPα [49]. The primary role for ATPα is to maintain cellular homeostasis across the axonal membrane of neurons. Although our data suggest that ATPα may act as a ubiquitin substrate *in vivo*, the downstream effects of this ubiquitin modification remain somewhat puzzling since there are no appreciable changes in ATPα stability of the protein by Western blot (**Figure 5**). Mutations in fly ATPα cause bang sensitivity, a phenotype analogous to seizures in mammals [50]. Seizures are a prominent characteristic of AS and occur in a substantial portion of 15 q duplication autism cases as well [1], [51]. In addition, increased α1 ATPase has recently been found in the axon initial segment of pyramidal and hippocampal neurons of *Ube3a* deficient mice [52]. Perhaps the most relevant connection between autism and UBE3A expression levels may be that increased activity of this Na+/K+ pump has been detected in the frontal cortex and cerebellum of individuals with idiopathic autism as compared to typically developing individuals [53]. Taken as a whole, these studies suggest that if Dube3a regulates the function of ATPα via direct ubiquitination these changes in Na+/K+ homeostasis may contribute to the increased seizure risk common in AS and autism.

We previously showed that Dube3a can regulate dopamine levels in the fly brain through the transcriptional (ubiquitin independent) regulation of GTP cyclohydrolase I (Punch) [25]. Although it had been known for some time that UBE3A is able to regulate steroid hormone receptors through transcriptional co-activation [54], this was the first time that transcriptionally regulated target of Dube3a was identified in the nervous system. Here we show that several other proteins are also regulated at the transcript level by Dube3a in a ubiquitination independent manner and have begun the search for transcription factors that may work in concert with Dube3a to regulate these genes (**Figure S1**). This growing list of transcriptionally regulated Dube3a genes opens a new avenue of research into the mechanisms by which Dube3a can act as part of a transcription factor complex and which transcription factors bind to Dube3a in the nucleus.

It should also be noted that we observed changes in the cellular compartmentalization of two proteins as a direct result of over-expression of either wild type Dube3a or the presumed dominant negative form Dube3a-C/A (Figure 2A). In the case of CG12140, the over-expression of wild type Dube3a caused a decrease in detectable levels of membrane bound CG12140 and an increase in the amount of CG12140 found in the nuclear fraction. For the protein Apolipophorins, there was a decrease in the cytoplasm and increase in the membrane bound levels of this protein when the Dube3a-C/A form was over-expressed. The trafficking of proteins to various cellular compartments as a result of ubiquitination or loss of ubiquitn moieties is not unprecedented. Reversible ubiquitination plays a key role in the modulation of the both Wnt and Hedgehog signaling pathways causing both the smoothend and Frizzled proteins to be rapidly recycled from the membrane to endosomes (reviewed in [55]). In fact, the Apolipophorin protein (also known as lipophorin or Retinoid- and fatty acid-binding glycoprotein) actually binds covalently to the Hedgehog morphogen and is required for long range Hedgehog signaling during development [56]. These findings suggest that Dube3a may even regulate some morphogens through the recycling of Apolipophorin to the membrane. Furthermore, in yeast, the ubiquitin ligase protein Rsp5 can “decorate” proteins with K63Ub moieties causing the subsequent movement of these proteins from the membrane to internal multivesicular bodies [57]. Although we provide no evidence that Dube3a is involved in these types of trafficking or signaling effects, it is nonetheless compelling to find these changes in cellular compartmental localization for at least two proteins identified in the screen and will be an interesting new avenue of Dube3a research in future studies.

Finally, although we have exhausted this particular approach to the identification of direct protein targets of Dube3a in fly heads, the use of the bio-Ub method will enable a deeper investigation into the identification of Dube3a dependent ubiquitin targets in the fly nervous system. Finding additional transcriptionally regulated Dube3a genes will require a different set of tools including whole genome chromatin immunoprecipitation approaches to identify the *cis* regulatory regions responsible for these changes in gene expression. Here we propose a new model for UBE3A regulation in the nervous system that involves not only regulation by direct ubiquitination of target proteins but also transcriptional regulation and perhaps even cellular trafficking.

## Supporting Information

Table S1 Complete list of proteins identified in our screen, fraction where they were identified, molecular weight of the protein excised from the gel, pH, fold change, CG identifying number and FBgn identifying number.
(PDF)

Table S2 Complete qRT-PCR data with error calculations from triplicate replicates for all genes identified in our screen. Genes with an asterisk (*) were used for transcription factor binding site analysis.
(PDF)

Table S3 DAVID analysis of proteins identified in the screen. Clusters of proteins that act in similar pathways or biological processes are listed along with an enrichment score for the cluster as well as a p_value_ and fold enrichment for a given group in the cluster as compared to the entire set of proteins.
(PDF)

File S1 This Microsoft PowerPoint file contains images of gels used for either direct comparison of experimental and control lanes or to excise a previously identified band for proteomic identification. The band numbers and gel numbers can be found in Table S1.
(PPTX)

## References

[1] Williams CA (2005) Neurological aspects of the Angelman syndrome. Brain Dev 27: 88–94. 15668046 10.1016/j.braindev.2003.09.014

[2] McKusick-Nathans Institute of Genetic Medicine (2012) Online Mendelian Inheritance in Man, OMIM®.

[3] Fang P, Lev-Lehman E, Tsai TF, Matsuura T, Benton CS, et al (1999) The spectrum of mutations in UBE3A causing Angelman syndrome. Hum Mol Genet 8: 129–135. 9887341 10.1093/hmg/8.1.129

[4] Kishino T, Lalande M, Wagstaff J (1997) UBE3A/E6-AP mutations cause Angelman syndrome. Nat Genet 15: 70–73. 8988171 10.1038/ng0197-70

[5] Matsuura T, Sutcliffe JS, Fang P, Galjaard RJ, Jiang YH, et al (1997) De novo truncating mutations in E6-AP ubiquitin-protein ligase gene (UBE3A) in Angelman syndrome. Nat Genet 15: 74–77. 8988172 10.1038/ng0197-74

[6] Hogart A, Wu D, Lasalle JM, Schanen NC (2010) The comorbidity of autism with the genomic disorders of chromosome 15q11.2-q13. Neurobiol Dis 38: 181–191. 18840528 10.1016/j.nbd.2008.08.011 PMC2884398

[7] Cook EH Jr, Lindgren V, Leventhal BL, Courchesne R, Lincoln A, et al (1997) Autism or atypical autism in maternally but not paternally derived proximal 15q duplication. Am J Hum Genet 60: 928–934. 9106540 PMC1712464

[8] Mao R, Jalal SM, Snow K, Michels VV, Szabo SM, et al (2000) Characteristics of two cases with dup(15)(q11.2-q12): one of maternal and one of paternal origin. Genetics in medicine : official journal of the American College of Medical Genetics 2: 131–135. 11397326 10.1097/00125817-200003000-00003

[9] Mohandas TK, Park JP, Spellman RA, Filiano JJ, Mamourian AC, et al (1999) Paternally derived de novo interstitial duplication of proximal 15 q in a patient with developmental delay. American journal of medical genetics 82: 294–300. 10051161

[10] Veltman MW, Thompson RJ, Craig EE, Dennis NR, Roberts SE, et al (2005) A paternally inherited duplication in the Prader-Willi/Angelman syndrome critical region: a case and family study. J Autism Dev Disord 35: 117–127. 15796127 10.1007/s10803-004-1039-1

[11] Smith SE, Zhou YD, Zhang G, Jin Z, Stoppel DC, et al (2011) Increased gene dosage of Ube3a results in autism traits and decreased glutamate synaptic transmission in mice. Science translational medicine 3: 103ra197. 10.1126/scitranslmed.3002627 PMC3356696 21974935

[12] Huibregtse JM, Scheffner M, Howley PM (1993) Cloning and expression of the cDNA for E6-AP, a protein that mediates the interaction of the human papillomavirus E6 oncoprotein with p53. Mol Cell Biol 13: 775–784. 8380895 10.1128/mcb.13.2.775-784.1993 PMC358960

[13] Hicke L (2001) Protein regulation by monoubiquitin. Nat Rev Mol Cell Biol 2: 195–201. 11265249 10.1038/35056583

[14] Cajigas IJ, Will T, Schuman EM (2010) Protein homeostasis and synaptic plasticity. The EMBO journal 29: 2746–2752. 20717144 10.1038/emboj.2010.173 PMC2924649

[15] Yashiro K, Riday TT, Condon KH, Roberts AC, Bernardo DR, et al (2009) Ube3a is required for experience-dependent maturation of the neocortex. Nature neuroscience 12: 777–783. 19430469 10.1038/nn.2327 PMC2741303

[16] Ehlers MD (2003) Activity level controls postsynaptic composition and signaling via the ubiquitin-proteasome system. Nat Neurosci 6: 231–242. 12577062 10.1038/nn1013

[17] Patrick GN, Bingol B, Weld HA, Schuman EM (2003) Ubiquitin-mediated proteasome activity is required for agonist-induced endocytosis of GluRs. Curr Biol 13: 2073–2081. 14653997 10.1016/j.cub.2003.10.028

[18] Sato M, Stryker MP (2010) Genomic imprinting of experience-dependent cortical plasticity by the ubiquitin ligase gene Ube3a. Proc Natl Acad Sci U S A 107: 5611–5616. 20212164 10.1073/pnas.1001281107 PMC2851788

[19] Jiang YH, Armstrong D, Albrecht U, Atkins CM, Noebels JL, et al (1998) Mutation of the Angelman ubiquitin ligase in mice causes increased cytoplasmic p53 and deficits of contextual learning and long-term potentiation. Neuron 21: 799–811. 9808466 10.1016/s0896-6273(00)80596-6

[20] Dindot SV, Antalffy BA, Bhattacharjee MB, Beaudet AL (2008) The Angelman syndrome ubiquitin ligase localizes to the synapse and nucleus, and maternal deficiency results in abnormal dendritic spine morphology. Hum Mol Genet 17: 111–118. 17940072 10.1093/hmg/ddm288

[21] Miura K, Kishino T, Li E, Webber H, Dikkes P, et al (2002) Neurobehavioral and electroencephalographic abnormalities in Ube3a maternal-deficient mice. Neurobiol Dis 9: 149–159. 11895368 10.1006/nbdi.2001.0463

[22] Greer PL, Hanayama R, Bloodgood BL, Mardinly AR, Lipton DM, et al (2010) The Angelman Syndrome protein Ube3A regulates synapse development by ubiquitinating arc. Cell 140: 704–716. 20211139 10.1016/j.cell.2010.01.026 PMC2843143

[23] Reiter LT, Seagroves TN, Bowers M, Bier E (2006) Expression of the Rho-GEF Pbl/ECT2 is regulated by the UBE3A E3 ubiquitin ligase. Hum Mol Genet 15: 2825–2835. 16905559 10.1093/hmg/ddl225 PMC3742451

[24] Margolis SS, Salogiannis J, Lipton DM, Mandel-Brehm C, Wills ZP, et al (2010) EphB-mediated degradation of the RhoA GEF Ephexin5 relieves a developmental brake on excitatory synapse formation. Cell 143: 442–455. 21029865 10.1016/j.cell.2010.09.038 PMC2967209

[25] Ferdousy F, Bodeen W, Summers K, Doherty O, Wright O, et al (2011) Drosophila Ube3a regulates monoamine synthesis by increasing GTP cyclohydrolase I activity via a non-ubiquitin ligase mechanism. Neurobiol Dis 41: 669–677. 21147225 10.1016/j.nbd.2010.12.001 PMC3040417

[26] Cummings ED, Brown JM, Sarva ST, Waldo RH, Hilliard GM (2007) High-throughput proteomics processing of proteins in polyacrylamide in a multiwell format. J Proteome Res 6: 1603–1608. 17367183 10.1021/pr060472y

[27] Wu Y, Bolduc FV, Bell K, Tully T, Fang Y, et al (2008) A Drosophila model for Angelman syndrome. Proc Natl Acad Sci U S A 105: 12399–12404. 18701717 10.1073/pnas.0805291105 PMC2527923

[28] Sullivan W, Ashburner M, Hawley RS (2000) Drosophila protocols. Cold Spring Harbor, N.Y.: Cold Spring Harbor Laboratory Press. xiv, 697 p. p.

[29] Mishra A, Godavarthi SK, Jana NR (2009) UBE3A/E6-AP regulates cell proliferation by promoting proteasomal degradation of p27. Neurobiol Dis 36: 26–34. 19591933 10.1016/j.nbd.2009.06.010

[30] Nasu J, Murakami K, Miyagawa S, Yamashita R, Ichimura T, et al (2010) E6AP ubiquitin ligase mediates ubiquitin-dependent degradation of peroxiredoxin 1. Journal of cellular biochemistry 111: 676–685. 20589759 10.1002/jcb.22752

[31] Pavlidis P, Noble WS (2003) Matrix2png: a utility for visualizing matrix data. Bioinformatics 19: 295–296. 12538257 10.1093/bioinformatics/19.2.295

[32] Joslyn CA, Mniszewski SM, Fulmer A, Heaton G (2004) The gene ontology categorizer. Bioinformatics 20 Suppl 1i169–177. 15262796 10.1093/bioinformatics/bth921

[33] Huang da W, Sherman BT, Lempicki RA (2009) Systematic and integrative analysis of large gene lists using DAVID bioinformatics resources. Nat Protoc 4: 44–57. 19131956 10.1038/nprot.2008.211

[34] Szklarczyk D, Franceschini A, Kuhn M, Simonovic M, Roth A, et al (2011) The STRING database in 2011: functional interaction networks of proteins, globally integrated and scored. Nucleic acids research 39: D561–568. 21045058 10.1093/nar/gkq973 PMC3013807

[35] Duffy JB (2002) GAL4 system in Drosophila: a fly geneticist’s Swiss army knife. Genesis 34: 1–15. 12324939 10.1002/gene.10150

[36] Franco M, Seyfried NT, Brand AH, Peng J, Mayor U (2011) A novel strategy to isolate ubiquitin conjugates reveals wide role for ubiquitination during neural development. Molecular & cellular proteomics : MCP 10: M110 002188. 10.1074/mcp.M110.002188 PMC3098581 20861518

[37] Tonon L, Touzet H, Varre JS (2010) TFM-Explorer: mining cis-regulatory regions in genomes. Nucleic Acids Res 38: W286–292. 20522509 10.1093/nar/gkq473 PMC2896114

[38] Jones BW, Fetter RD, Tear G, Goodman CS (1995) glial cells missing: a genetic switch that controls glial versus neuronal fate. Cell 82: 1013–1023. 7553843 10.1016/0092-8674(95)90280-5

[39] Viktorin G, Riebli N, Popkova A, Giangrande A, Reichert H (2011) Multipotent neural stem cells generate glial cells of the central complex through transit amplifying intermediate progenitors in Drosophila brain development. Developmental biology 356: 553–565. 21708145 10.1016/j.ydbio.2011.06.013

[40] McQuilton P, St Pierre SE, Thurmond J (2012) FlyBase 101–the basics of navigating FlyBase. Nucleic acids research 40: D706–714. 22127867 10.1093/nar/gkr1030 PMC3245098

[41] Kao WH, Beaudenon SL, Talis AL, Huibregtse JM, Howley PM (2000) Human papillomavirus type 16 E6 induces self-ubiquitination of the E6AP ubiquitin-protein ligase. J Virol 74: 6408–6417. 10864652 10.1128/jvi.74.14.6408-6417.2000 PMC112148

[42] Kishino T, Lalande M, Wagstaff J (1997) UBE3A/E6-AP mutations cause Angelman syndrome [published erratum appears in Nat Genet 1997 Apr;15(4): 411]. Nat Genet 15: 70–73. 8988171 10.1038/ng0197-70

[43] Wang CL, Coluccio LM (2010) New insights into the regulation of the actin cytoskeleton by tropomyosin. International review of cell and molecular biology 281: 91–128. 20460184 10.1016/S1937-6448(10)81003-2 PMC2923581

[44] Kim D, Kim SH, Li GC (1999) Proteasome inhibitors MG132 and lactacystin hyperphosphorylate HSF1 and induce hsp70 and hsp27 expression. Biochem Biophys Res Commun 254: 264–268. 9920768 10.1006/bbrc.1998.9840

[45] Mathew A, Mathur SK, Morimoto RI (1998) Heat shock response and protein degradation: regulation of HSF2 by the ubiquitin-proteasome pathway. Mol Cell Biol 18: 5091–5098. 9710593 10.1128/mcb.18.9.5091 PMC109094

[46] Kim SA, Yoon JH, Kim DK, Kim SG, Ahn SG (2005) CHIP interacts with heat shock factor 1 during heat stress. FEBS Lett 579: 6559–6563. 16293251 10.1016/j.febslet.2005.10.043

[47] Mishra A, Godavarthi SK, Maheshwari M, Goswami A, Jana NR (2009) The ubiquitin ligase E6-AP is induced and recruited to aggresomes in response to proteasome inhibition and may be involved in the ubiquitination of Hsp70-bound misfolded proteins. The Journal of biological chemistry 284: 10537–10545. 19233847 10.1074/jbc.M806804200 PMC2667740

[48] Ballinger CA, Connell P, Wu Y, Hu Z, Thompson LJ, et al (1999) Identification of CHIP, a novel tetratricopeptide repeat-containing protein that interacts with heat shock proteins and negatively regulates chaperone functions. Mol Cell Biol 19: 4535–4545. 10330192 10.1128/mcb.19.6.4535 PMC104411

[49] Lebovitz RM, Takeyasu K, Fambrough DM (1989) Molecular characterization and expression of the (Na[+]+K[+])-ATPase -subunit in Drosophila melanogaster. The EMBO journal 8: 193–202. 2540956 10.1002/j.1460-2075.1989.tb03364.x PMC400789

[50] Schubiger M, Feng Y, Fambrough DM, Palka J (1994) A mutation of the Drosophila sodium pump subunit gene results in bang-sensitive paralysis. Neuron 12: 373–381. 8110464 10.1016/0896-6273(94)90278-x

[51] Dennis NR, Veltman MW, Thompson R, Craig E, Bolton PF, et al (2006) Clinical findings in 33 subjects with large supernumerary marker(15) chromosomes and 3 subjects with triplication of 15q11-q13. Am J Med Genet A 140: 434–441. 16470730 10.1002/ajmg.a.31091

[52] Kaphzan H, Buffington SA, Jung JI, Rasband MN, Klann E (2011) Alterations in intrinsic membrane properties and the axon initial segment in a mouse model of angelman syndrome. The Journal of neuroscience : the official journal of the Society for Neuroscience 31: 17637–17648. 22131424 10.1523/JNEUROSCI.4162-11.2011 PMC3483031

[53] Ji L, Chauhan A, Brown WT, Chauhan V (2009) Increased activities of Na+/K+-ATPase and Ca2+/Mg2+-ATPase in the frontal cortex and cerebellum of autistic individuals. Life sciences 85: 788–793. 19863947 10.1016/j.lfs.2009.10.008 PMC2810556

[54] Ramamoorthy S, Nawaz Z (2008) E6-associated protein (E6-AP) is a dual function coactivator of steroid hormone receptors. Nucl Recept Signal 6: e006. 18432313 10.1621/nrs.06006 PMC2329825

[55] Clague MJ, Liu H, Urbe S (2012) Governance of endocytic trafficking and signaling by reversible ubiquitylation. Dev Cell 23: 457–467. 22975321 10.1016/j.devcel.2012.08.011

[56] Panakova D, Sprong H, Marois E, Thiele C, Eaton S (2005) Lipoprotein particles are required for Hedgehog and Wingless signalling. Nature 435: 58–65. 15875013 10.1038/nature03504

[57] Erpapazoglou Z, Dhaoui M, Pantazopoulou M, Giordano F, Mari M, et al (2012) A dual role for K63-linked ubiquitin chains in multivesicular body biogenesis and cargo sorting. Mol Biol Cell 23: 2170–2183. 22493318 10.1091/mbc.E11-10-0891 PMC3364180

