# Supplementary material for: Proteomic Profiling in Drosophila Reveals Potential Dube3a Regulation of the Actin Cytoskeleton and Neuronal Homeostasis
Source: PLoS One. 2013 Apr 23;8(4):e61952. doi: 10.1371/journal.pone.0061952 (PMC3633955; doi:10.1371/journal.pone.0061952)
Supplement: Table S1 — Complete list of proteins identified in our screen, fraction where they were identified, molecular weight of the protein excised from the gel, pH, fold change, CG identifying number and FBgn identifying number. (PDF) [file pone.0061952.s001.pdf]

**Supplemental Table 1**

| Gene Name | Extract | MW | pH | Fc | Fc in | Slide # in File | Band # in slide | CG number | FBgn number |
|---|---|---|---|---|---|---|---|---|---|
| Actin 57B | CE | 27.750 | 6.23 | +2.51 | HS>Dube3a-C/A | 2 | 5 | CG10067 | FBgn0000044 |
| | CE | 40.150 | 6.35 | +2.05 | HS>Dube3a | 3 | 7 | | |
| Actin-5c | ME | 59.742 | 7.78 | +66.37 | HS>Dube3a | 7 | 24 | CG4027 | FBgn0000042 |
| | ME | 60.949 | 7.78 | +27.17 | $Dube3a^{15b}$ | 9 | 28 | | |
| | CE | 41.736 | 5.46 | +4.32 | hUBE3a-77L | 1 | 5 | | |
| | CE | 41.457 | 5.86 | +2.28 | hUBE3a-77L | 1 | 6 | | |
| | ME | 163.810 | 7.12 | -3.86 | HS>Dube3a | 7 | 10 | | |
| Alcohol Dehydrogenase | CE | 26.830 | 7.12 | +4.35 | HS>Dube3a | 3 | 19 | CG3481 | FBgn0000055 |
| | ME | 68.987 | 7.18 | -2.87 | $Dube3a^{15b}$ | 10 | 17 | | |
| | ME | 25.458 | 7.46 | -3.84 | HS>Dube3a | 7 | 23 | | |
| | ME | 38.859 | 7.12 | -6.22 | $Dube3a^{15b}$ | 10 | 16 | | |
| Aldehyde Dehydrogenase | CE | 52.887 | 6.22 | +6.64 | hUBE3a-77L | 1 | 11 | CG3752 | FBgn0012036 |
| | CE | 40.675 | 6.83 | +4.92 | HS>Dube3a-C/A | 2 | 13 | | |
| | ME | 50.620 | 6.12 | +3.19 | $Dube3a^{15b}$ | 10 | 8 | | |
| Aldolase | CE | 37.452 | 7.78 | +34.48 | HS>Dube3a-C/A | 2 | 23 | CG6058 | FBgn0000064 |
| | CE | 40.260 | 7.12 | +24.84 | HS>Dube3a | 3 | 16 | | |
| | CE | 29.340 | 7.67 | +2.04 | HS>Dube3a | 3 | 25 | | |
| | ME | 39.436 | 7.46 | -3.05 | $Dube3a^{15b}$ | 9 | 25 | | |
| | ME | 27.382 | 7.12 | -7.71 | HS>Dube3a-C/A | 8 | 16 | | |
| Alpha-Tubulin | CE | 51.910 | 6.22 | 4.17 | hUBE3a-77L | 1 | 12 | CG1913 | FBgn0003884 |
| Apolipophorins | ME | 61.646 | 8.38 | +33.05 | HS>Dube3a-C/A | 8 | 28 | CG11064 | FBgn0087002 |
| | CE | 228.690 | 7.41 | +5.88 | HS>Dube3a | 3 | 21 | | |
| | CE | 60.511 | 8.55 | -390.23 | HS>Dube3a-C/A | 2 | 25 | | |
| Arginine Kinase | CE | 38.580 | 7.12 | +110.12 | HS>Dube3a | 3 | 17 | CG32031 | FBgn0000116 |
| | CE | 37.530 | 6.76 | +32.52 | HS>Dube3a | 3 | 10 | | |
| | CE | 36.730 | 7.12 | +10.01 | HS>Dube3a | 3 | 18 | | |
| | NE | 38.387 | 6.84 | +2.46 | HS>Dube3a | 6 | 12 | | |
| | ME | 38.051 | 6.33 | -2.72 | $Dube3a^{15b}$ | 10 | 4 | | |
| | ME | 38.204 | 6.69 | -3.87 | HS>Dube3a-C/A | 8 | 10 | | |
| | ME | 37.260 | 6.69 | -4.31 | $Dube3a^{15b}$ | 10 | 10 | | |
| | ME | 37.510 | 6.69 | -5.85 | HS>Dube3a | 7 | 7 | | |
| | CE | 24.390 | 6.53 | -6.66 | HS>Dube3a-C/A | 2 | 6 | | |
| | CE | 37.440 | 6.53 | -8.11 | HS>Dube3a | 3 | 4 | | |
| ATP synthase-β | CE | 52.890 | 4.8 | +3.97 | hUBE3a-77L | 1 | 1 | CG11154 | FBgn0010217 |

| | | | | | | | | | |
|---|---|---|---|---|---|---|---|---|---|
| ATPalpha | ME | 60.577 | 6.12 | +103.32 | *Dube3a*[15b] | 10 | 7 | CG5670 | FBgn0002921 |
| | CE | 69.420 | 6.35 | +18.06 | HS>Dube3a | 3 | 6 | | |
| | ME | 60.028 | 6.1 | +17 | HS>Dube3a-C/A | 8 | 5 | | |
| | CE | 91.060 | 6.53 | +2.62 | HS>Dube3a-C/A | 2 | 8 | | |
| | CE | 33.120 | 6.14 | +2.35 | HS>Dube3a | 3 | 3 | | |
| | ME | 95.858 | 6.33 | -3 | HS>Dube3a | 7 | 1 | | |
| | CE | 93.860 | 6.15 | -5.56 | HS>Dube3a | 3 | 1 | | |
| Bellwether | CE | 47.862 | 8.24 | +8.94 | HS>Dube3a-C/A | 2 | 24 | CG3612 | FBgn0011211 |
| | ME | 53.412 | 8.01 | +5.63 | *Dube3a*[15b] | 9 | 29 | | |
| | ME | 52.414 | 8.24 | +5.35 | *Dube3a*[15b] | 9 | 31 | | |
| | ME | 52.538 | 7.36 | +2.26 | *Dube3a*[15b] | 10 | 23 | | |
| | ME | 52.538 | 7.26 | +2.26 | *Dube3a*[15b] | 10 | 19 | | |
| | ME | 54.332 | 8.38 | +2.15 | HS>Dube3a-C/A | 8 | 34 | | |
| CG12140 | CE | 68.300 | 6.22 | +27.67 | hUBE3a-77L | 1 | 10 | CG12140 | FBgn0033465 |
| | NE | 70.320 | 6.43 | +23.61 | HS>Dube3a | 6 | 11 | | |
| | ME | 67.300 | 6.33 | -10.78 | *Dube3a*[15b] | 10 | 3 | | |
| | ME | 67.269 | 6.33 | -53.92 | HS>Dube3a | 7 | 3 | | |
| CG3415 (Mfe2) | ME | 62.057 | 6.97 | +9.88 | HS>Dube3a-C/A | 8 | 18 | CG3415 | FBgn0030731 |
| CG3731 | NE | 46.374 | 6.05 | +5.01 | *Dube3a*[15b] | 5 | 20 | CG3731 | FBgn0038271 |
| | NE | 46.374 | 6.07 | +3.27 | hUBE3a-77L | 5 | 18 | | |
| CG6045 | CE | 40.170 | 6.23 | +5.76 | HS>Dube3a-C/A | 2 | 3 | CG6045 | FBgn0038349 |
| CG7430 | CE | 167.620 | 6.23 | +3256 | HS>Dube3a-C/A | 2 | 1 | CG7430 | FBgn0036762 |
| | CE | 53.070 | 7.12 | +8.24 | HS>Dube3a | 3 | 14 | | |
| | CE | 51.590 | 6.76 | +5.33 | HS>Dube3a | 3 | 8 | | |
| | CE | 51.380 | 6.14 | +3.36 | HS>Dube3a | 3 | 2 | | |
| CG7461 | ME | 63.310 | 7.79 | +21.33 | HS>Dube3a-C/A | 8 | 27 | CG7461 | FBgn0034432 |
| CG7920 | CE | 48.940 | 7.92 | +3.25 | HS>Dube3a | 3 | 27 | CG7920 | FBgn0039737 |
| Chaoptin | CE | 144.040 | 7.27 | +28.36 | HS>Dube3a-C/A | 2 | 17 | CG1744 | FBgn0000313 |
| | ME | 175.419 | 7.6 | +8.38 | HS>Dube3a-C/A | 8 | 25 | | |
| | NE | 166.721 | 7.27 | +5.16 | *Dube3a*[15b] | 4 | 1 | | |
| | NE | 157.000 | 6.91 | +3.2 | *Dube3a*[15b] | 5 | 28 | | |
| Crystallin | ME | 58.770 | 7.29 | +33.52 | HS>Dube3a | 7 | 17 | CG16963 | FBgn0005664 |
| | NE | 70.856 | 5.46 | +7.71 | HS>Dube3a | 6 | 3 | | |
| | NE | 55.982 | 6.07 | +2.36 | hUBE3a-77L | 5 | 16 | | |
| Cuticular protein 72Ec | NE | 47.553 | 6.84 | +111.34 | hUBE3a-77L | 5 | 27 | CG4784 | FBgn0036619 |
| | NE | 43.555 | 6.51 | +94.95 | *Dube3a*[15b] | 5 | 22 | | |
| | NE | 44.382 | 5.81 | +31.37 | *Dube3a*[15b] | 5 | 9 | | |
| | NE | 43.147 | 6.05 | +18.77 | *Dube3a*[15b] | 5 | 21 | | |
| | NE | 47.852 | 6.07 | +15.21 | hUBE3a-77L | 5 | 17 | | |
| | NE | 43.578 | 7.89 | +8.62 | *Dube3a*[15b] | 4 | 7 | | |
| | NE | 50.000 | 5.58 | +3.87 | hUBE3a-77L | 5 | 6 | | |

| | | | | | | | | | |
|---|---|---|---|---|---|---|---|---|---|
| | CE | 50.630 | 5.46 | +2.64 | hUBE3a-77L | 1 | 4 | | |
| Dynamin | NE | 94.703 | 7.46 | +2.61 | *Dube3a*$^{15b}$ | 4 | 2 | CG18102 | FBgn0003392 |
| Enolase | CE | 47.470 | 7.12 | +7.28 | HS>Dube3a | 3 | 15 | CG17654 | FBgn0000579 |
| | CE | 21.560 | 6.53 | -2.6 | HS>Dube3a-C/A | 2 | 7 | | |
| | ME | 46.293 | 6.69 | -3.3 | *Dube3a*$^{15b}$ | 10 | 9 | | |
| | ME | 47.502 | 6.69 | -3.68 | HS>Dube3a-C/A | 8 | 9 | | |
| | ME | 46.853 | 6.69 | -4.05 | Dube3a | 7 | 6 | | |
| | ME | 45.970 | 7.26 | -10.59 | *Dube3a*$^{15b}$ | 10 | 20 | | |
| | ME | 45.970 | 7.12 | -11.19 | *Dube3a*$^{15b}$ | 10 | 15 | | |
| Eps15 | CE | 96.770 | 5.46 | +2.66 | hUBE3a-77L | 1 | 2 | CG16932 | FBgn0035060 |
| ERp60 | CE | 43.749 | 6.53 | +5.91 | Dube3a-C/A | 2 | 11 | CG8983 | FBgn0033663 |
| Failed Axon Connections | CE | 62.200 | 5.45 | +10.75 | *Dube3a*$^{15b}$ | 11 | 1 | CG4609 | FBgn0014163 |
| Fasciclin 1 | ME | 60.950 | 7.36 | +11 | *Dube3a*$^{15b}$ | 10 | 22 | CG6588 | FBgn0262742 |
| Fat-body protein 1 | NE | 47.550 | 6.91 | +60.64 | *Dube3a*$^{15b}$ | 5 | 29 | CG17285 | FBgn0000639 |
| Fimbrin | CE | 70.910 | 5.94 | +102.3 | *Dube3a*$^{15b}$ | 11 | 3 | CG8649 | FBgn0024238 |
| glyceraldehyde 3 p | CE | 35.610 | 8.45 | +2.64 | Dube3a-C/A | 2 | 28 | CG12055 | FBgn0001091 |
| Glycogen Phosphorylase | CE | 84.490 | 7.27 | +7.54 | Dube3a-C/A | 2 | 18 | CG7254 | FBgn0004507 |
| HSP 70Ab | CE | 72.240 | 6.22 | +8.37 | hUBE3a-77L | 1 | 9 | CG18743 | FBgn0013276 |
| HSP 70Ba | CE | 58.040 | 6.83 | +30.95 | Dube3a-C/A | 2 | 12 | CG31449 | FBgn0013277 |
| | ME | 71.620 | 6.33 | -6.19 | HS>Dube3a | 7 | 2 | | |
| | ME | | 6.33 | -14.53 | *Dube3a*$^{15b}$ | 10 | 2 | | |
| HSP 70Bb | CE | 56.330 | 6.53 | +3.72 | Dube3a-C/A | 2 | 10 | CG31359 | FBgn0013278 |
| HSP cognate 2 | CE | 63.910 | 6.23 | +5.46 | Dube3a-C/A | 2 | 2 | CG7756 | FBgn0001217 |
| | CE | 73.240 | 6.35 | +4.69 | HS>Dube3a | 3 | 5 | | |
| HSP cognate 4 | CE | 35.610 | 7.27 | +26.04 | Dube3a-C/A | 2 | 21 | CG4264 | FBgn0001219 |
| | CE | 71.800 | 5.46 | +2.35 | hUBE3a-77L | 1 | 3 | | |
| Malic Enzyme | ME | 60.940 | 6.58 | +5.52 | HS>Dube3a | 7 | 9 | CG10120 | FBgn0002719 |
| Myosin Heavy Chain | NE | 71.666 | 6.02 | +13.35 | HS>Dube3a | 6 | 6 | CG17927 | FBgn0086783 |
| | CE | 254.443 | 6.22 | +7.42 | hUBE3a-77L | 1 | 7 | | |
| | ME | | 8.24 | +7.34 | *Dube3a*$^{15b}$ | 9 | 30 | | |
| | NE | 41.181 | 6.02 | +4.27 | HS>Dube3a | 6 | 9 | | |
| | NE | 250.000 | 6.07 | +3.5 | hUBE3a-77L | 5 | 13 | | |
| | NE | 250.000 | 6.05 | +2.89 | *Dube3a*$^{15b}$ | 5 | 19 | | |
| | NE | 238.130 | 5.85 | -5.25 | HS>Dube3a | 6 | 1 | | |
| NADH-ubiquinone oxidoreductase 75 kDa subunit | ME | 75.264 | 6.55 | +5.38 | *Dube3a*$^{15b}$ | 10 | 11 | CG2286 | FBgn0017566 |

| | | | | | | | | | |
|---|---|---|---|---|---|---|---|---|---|
| | ME | 77.254 | 6.33 | -6.66 | Dube3a-C/A | 8 | 2 | | |
| neither inactivation nor | CE | 134.820 | 6.22 | +10.91 | hUBE3a-77L | 1 | 8 | CG5125 | FBgn0002938 |
| Phosphoglycerate Kinase | CE | 46.490 | 7.67 | +1.78 | HS>Dube3a | 3 | 23 | CG3127 | FBgn0250906 |
| Porin | CE | 23.670 | 7.2 | -5.6 | Dube3a-C/A | 2 | 16 | CG6647 | FBgn0004363 |
| Prolyl endopeptidase | CE | 86.410 | 5.94 | +16.76 | *Dube3a*$^{15b}$ | 11 | 2 | CG5355 | FBgn0032242 |
| Pro-phenol Oxidase 1 | CE | 80.500 | 6.69 | 3.35 | Dube3a15b | 11 | 4 | CG42639 | FBgn0261382 |
| | ME | 68.990 | 7.12 | -2.87 | *Dube3a*$^{15b}$ | 10 | 14 | CG42639 | FBgn0261362 |
| Protein disulfide-isomerase | NE | 51.150 | 6.02 | +2.44 | HS>Dube3a | 6 | 8 | CG6988 | FBgn0014002 |
| Punch | ME | 32.270 | 6.33 | -9.39 | *Dube3a*$^{15b}$ | 10 | 5 | CG9441 | FBgn0003162 |
| Regucalcin | CE | 24.399 | 6.83 | +14.04 | Dube3a-C/A | 2 | 15 | CG1803 | FBgn0030362 |
| Transferrin 1 | CE | 57.009 | 7.27 | +22.01 | Dube3a-C/A | 2 | 20 | CG6186 | FBgn0022355 |
| | CE | 70.790 | 7.12 | +16.52 | HS>Dube3a | 3 | 13 | | |
| Tropomyosin I | NE | 36.750 | 5.58 | +2.2 | hUBE3a-77L | 5 | 8 | CG4898 | FBgn0003721 |
| Unidentified | NE | 41.900 | 7.46 | +2.29 | *Dube3a*$^{15b}$ | 4 | 4 | | |
