# Supplementary material for: Proteomic Profiling in Drosophila Reveals Potential Dube3a Regulation of the Actin Cytoskeleton and Neuronal Homeostasis
Source: PLoS One. 2013 Apr 23;8(4):e61952. doi: 10.1371/journal.pone.0061952 (PMC3633955; doi:10.1371/journal.pone.0061952)
Supplement: Table S2 — Complete qRT-PCR data with error calculations from triplicate replicates for all genes identified in our screen. Genes with an asterisk (*) were used for transcription factor binding site analysis. (PDF) [file pone.0061952.s002.pdf]

Supplemental Table 2

Gene	*Dube3a^{15b}*	HS>Dube3a	HS>Dube3a-C/A
Actin 57B	1.62 ± 0.08	-1.07 ± 0.07	-1.22 ± 0.06
Actin-5c	-1.19 ± 0.13	1.09 ± 0.01	-1.27 ± 0.1
Alcohol Dehydrogenase	-1.30 ± 0.07	-1.07 ± 0.07	1.27 ± 0.13
Aldehyde Dehydrogenase	-1.38 ± 0.18	1.52 ± 0.12	1.36 ± 0.11
Aldolase	-1.09 ± 0.06	-1.17 ± 0.10	-1.39 ± 0.15
Alpha-Tubulin	1.08 ± 0.06	1.12 ± 0.10	1.31 ± 0.04
*Apolipophorins	1.43 ± 0.08	1.07 ± 0.01	2.20 ± 0.06
Arginine Kinase set 1	-1.17 ± 0.03	-1.36 ± 0.07	-1.40 ± 0.26
Arginine Kinase set 2	-1.32 ± 0.08	-1.20 ± 0.06	-1.28 ± 0.09
ATP synthase-β	-1.75 ± 0.13	-1.43 ± 0.06	-1.79 ± 0.05
ATPα	1.07 ± 0.01	-1.30 ± 0.06	-1.31 ± 0.24
Bellwether	-1.51 ± 0.06	-1.05 ± 0.02	-1.53 ± 0.44
CG12140	-1.46 ± 0.03	-1.14 ± 0.05	-1.21 ± 0.21
CG3415 (Mfe2)	1.10 ± 0.07	-1.12 ± 0.04	-1.23 ± 0.06
CG3731	-1.60 ± 0.17	-1.14 ± 0.12	-1.38 ± 0.07
CG6045	1.44 ± 0.08	1.27 ± 0.17	-1.11 ± 0.05
CG7430	1.38 ± 0.03	-1.05 ± 0.05	-1.81 ± 0.36
CG7461	-1.33 ± 0.20	-1.11 ± 0.07	-1.23 ± 0.14
CG7920		2.54 ± 0.18	3.87 ± 0.11
Chaoptin	1.23 ± 0.04	-1.09 ± 0.07	-1.29 ± 0.16
*Crystallin	-1.78 ± 0.33	2.08 ± 0.20	2.20 ± 0.32
Cuticular protein 72Ec	1.59 ± 0.05	2.50 ± 0.15	6.34 ± 0.47
Dynamin	1.99 ± 0.06	1.06 ± 0.05	1.49 ± 0.28
Enolase	-1.28 ± 0.10	-2.49 ± 0.31	-2.79 ± 0.71
*Eps15	-1.33 ± 0.14	1.27 ± 0.24	1.62 ± 0.54
ERp60	1.11 ± 0.07	-1.61 ± 0.12	-1.73 ± 0.08
Failed Axon Connections	2.75 ± 0.39	-1.28 ± 0.06	1.33 ± 0.31
*Fasciclin 1	1.39 ± 0.10	1.88 ± 0.46	1.87 ± 0.13
*Fat-body protein 1	-3.32 ± 0.46	2.08 ± 0.93	1.76 ± 0.13
Fimbrin	1.27 ± 0.11	1.09 ± 0.03	1.13 ± 0.04
*GAPDH	1.19 ± 0.14	1.77 ± 0.31	1.87 ± 0.25
Glycogen Phosphorylase	-2.32 ± 0.10	-2.74 ± 0.13	-1.38 ± 0.07
HSP 70Ab	25.23 ± 1.87	2.90 ± 0.29	2.56 ± 0.22
HSP 70Ba	27.50 ± 1.09	2.99 ± 0.25	2.98 ± 0.26
HSP 70Bb	60.63 ± 27.4	2.58 ± 0.15	2.87 ± 0.65
HSP cognate 2	1.28 ± 0.15	1.32 ± 0.09	-1.36 ± 0.25
HSP cognate 4	2.67 ± 0.42	1.17 ± 0.05	-1.59 ± 0.16
Malic Enzyme	-1.41 ± 0.10	1.19 ± 0.02	1.14 ± 0.01
Myosin Heavy Chain set 1	5.34 ± 0.23	-1.43 ± 0.05	-2.16 ± 0.40
Myosin Heavy Chain set 2	-1.55 ± 0.04	1.22 ± 0.09	-1.64 ± 0.06
NADH	-1.06 ± 0.02	-2.28 ± 0.08	-5.06 ± 0.94
*neither inactivation nor afterpotential C	1.05 ± 0.05	2.05 ± 0.13	2.48 ± 0.25
Phosphoglycerate Kinase	-1.37 ± 0.08	-1.72 ± 0.20	1.12 ± 0.11
Porin	-1.97 ± 0.26	-1.28 ± 0.23	-1.20 ± 0.11
Pro-phenol Oxidase 1			
Prolyl endopeptidase	1.33 ± 0.17	1.36 ± 0.22	1.36 ± 0.17
Protein disulfide-isomerase	2.39 ± 0.33	1.30 ± 0.11	1.40 ± 0.06
*Punch	-1.51 ± 0.19	2.03 ± 0.50	2.96 ± 0.60
Regucalcin	-1.04 ± 0.02	-1.66 ± 0.20	-3.23 ± 0.27
Transferrin 1	-1.19 ± 0.06	1.24 ± 0.08	1.12 ± 0.09
Tropomyosin I	-3.25 ± 0.49	-1.41 ± 0.04	-1.89 ± 0.07
