# Supplementary material for: Proteomic Profiling in Drosophila Reveals Potential Dube3a Regulation of the Actin Cytoskeleton and Neuronal Homeostasis
Source: PLoS One. 2013 Apr 23;8(4):e61952. doi: 10.1371/journal.pone.0061952 (PMC3633955; doi:10.1371/journal.pone.0061952)
Supplement: Table S3 — DAVID analysis of proteins identified in the screen. Clusters of proteins that act in similar pathways or biological processes are listed along with an enrichment score for the cluster as well as a pvalue and fold enrichment for a given group in the cluster as compared to the entire set of proteins. (PDF) [file pone.0061952.s003.pdf]

Supplemental Table 3

Category	Term	Count	%	F_E	P_{Value}	Benjamini
**Cluster 1**	**Enrichment Score: 4.1**					
SP_PIR_KEYWORDS	ATP	6	14	26.6	2.488E-06	1.31E-04
SP_PIR_KEYWORDS	nucleotide-binding	13	30	5.3	2.549E-06	8.92E-05
GOTERM_MF_FAT	GO:0000166~nucleotide binding	19	44	3.1	4.379E-06	7.00E-04
SP_PIR_KEYWORDS	atp-binding	11	26	5.7	1.275E-05	3.35E-04
GOTERM_MF_FAT	GO:0017076~purine nucleotide binding	16	37	3.2	3.434E-05	2.74E-03
GOTERM_MF_FAT	GO:0030554~adenyl nucleotide binding	14	33	3.4	8.096E-05	4.31E-03
GOTERM_MF_FAT	GO:0001883~purine nucleoside binding	14	33	3.4	8.630E-05	3.45E-03
GOTERM_MF_FAT	GO:0001882~nucleoside binding	14	33	3.4	9.428E-05	3.01E-03
GOTERM_MF_FAT	GO:0032555~purine ribonucleotide binding	13	30	2.8	1.142E-03	2.26E-02
GOTERM_MF_FAT	GO:0032553~ribonucleotide binding	13	30	2.8	1.142E-03	2.26E-02
GOTERM_MF_FAT	GO:0005524~ATP binding	11	26	2.9	2.681E-03	4.66E-02
GOTERM_MF_FAT	GO:0032559~adenyl ribonucleotide binding	11	26	2.9	2.734E-03	4.29E-02
**Cluster 2**	**Enrichment Score: 3.8**					
SP_PIR_KEYWORDS	ATP	6	14	26.6	2.488E-06	1.31E-04
INTERPRO	IPR018181:Heat shock protein 70, conserved site	4	9	80.9	1.295E-05	1.77E-03
INTERPRO	IPR013126:Heat shock protein 70	4	9	74.7	1.679E-05	1.15E-03
INTERPRO	IPR001023:Heat shock protein Hsp70	4	9	74.7	1.679E-05	1.15E-03
SP_PIR_KEYWORDS	stress response	4	9	52.5	5.333E-05	9.33E-04
PIR_SUPERFAMILY	PIRSF002581:chaperone HSP70	4	9	38.5	1.022E-04	2.55E-03
SP_PIR_KEYWORDS	molecular chaperone	3	7	129.4	2.125E-04	2.03E-03
GOTERM_BP_FAT	GO:0009266~response to temperature stimulus	5	12	13.6	4.247E-04	4.07E-02
GOTERM_BP_FAT	GO:0009628~response to abiotic stimulus	6	14	5.6	3.710E-03	1.41E-01
GOTERM_BP_FAT	GO:0009408~response to heat	4	9	11.7	4.410E-03	1.53E-01
KEGG_PATHWAY	dme04144:Endocytosis	5	12	6.1	6.423E-03	9.21E-02
**Cluster 3**	**Enrichment Score: 3.7**					
GOTERM_BP_FAT	GO:0006091~generation of precursor metabolites and energy	10	23	9.1	7.510E-07	3.67E-04
KEGG_PATHWAY	dme00010:Glycolysis / Gluconeogenesis	7	16	13.2	5.805E-06	1.74E-04
SP_PIR_KEYWORDS	glycolysis	4	9	50.3	6.082E-05	9.12E-04
GOTERM_BP_FAT	GO:0044275~cellular carbohydrate catabolic process	5	12	19.5	1.054E-04	2.54E-02
GOTERM_BP_FAT	GO:0046164~alcohol catabolic process	5	12	19.5	1.054E-04	2.54E-02
GOTERM_BP_FAT	GO:0006006~glucose metabolic process	5	12	16.3	2.124E-04	2.56E-02
SP_PIR_KEYWORDS	gluconeogenesis	3	7	129.4	2.125E-04	2.03E-03
UP_SEQ_FEATURE	binding site:Substrate	5	12	12.4	5.004E-04	2.98E-02
GOTERM_BP_FAT	GO:0006096~glycolysis	4	9	22.7	6.440E-04	5.11E-02
GOTERM_BP_FAT	GO:0016052~carbohydrate catabolic process	5	12	12.0	6.922E-04	4.72E-02
GOTERM_BP_FAT	GO:0019318~hexose metabolic process	5	12	11.7	7.573E-04	4.53E-02
GOTERM_BP_FAT	GO:0006007~glucose catabolic process	4	9	18.5	1.182E-03	6.22E-02
GOTERM_BP_FAT	GO:0019320~hexose catabolic process	4	9	18.5	1.182E-03	6.22E-02
GOTERM_BP_FAT	GO:0005996~monosaccharide metabolic process	5	12	10.2	1.242E-03	5.90E-02
GOTERM_BP_FAT	GO:0046365~monosaccharide catabolic process	4	9	18.0	1.264E-03	5.47E-02
**Cluster 4**	**Enrichment Score: 3.3**					
SP_PIR_KEYWORDS	oxidoreductase	12	28	6.5	1.125E-06	1.18E-04
GOTERM_BP_FAT	GO:0055114~oxidation reduction	12	28	3.8	1.338E-04	2.16E-02
GOTERM_MF_FAT	GO:0048037~cofactor binding	7	16	7.5	2.576E-04	6.85E-03
GOTERM_MF_FAT	GO:0050662~coenzyme binding	6	14	8.5	5.526E-04	1.26E-02
GOTERM_MF_FAT	GO:0051287~NAD or NADH binding	3	7	14.8	1.639E-02	1.98E-01
GOTERM_MF_FAT	GO:0050660~FAD binding	3	7	9.7	3.605E-02	3.07E-01
**Cluster 5**	**Enrichment Score: 2.9**					
SP_PIR_KEYWORDS	cytoplasm	9	21	6.2	6.569E-05	8.62E-04
SP_PIR_KEYWORDS	cytoskeleton	4	9	13.6	2.949E-03	2.19E-02
GOTERM_CC_FAT	GO:0015629~actin cytoskeleton	4	9	8.1	1.115E-02	3.15E-01
**Cluster 6**	**Enrichment Score: 2.1**					
SP_PIR_KEYWORDS	Redox-active center	3	7	60.4	1.045E-03	8.41E-03
GOTERM_BP_FAT	GO:0042592~homeostatic process	5	12	6.1	8.060E-03	2.46E-01
GOTERM_BP_FAT	GO:0019725~cellular homeostasis	4	9	7.2	1.689E-02	4.26E-01
GOTERM_BP_FAT	GO:0045454~cell redox homeostasis	3	7	11.2	2.769E-02	4.97E-01
**Cluster 7**	**Enrichment Score: 1.8**					
SP_PIR_KEYWORDS	actin-binding	3	7	19.7	9.668E-03	6.57E-02
GOTERM_MF_FAT	GO:0008092~cytoskeletal protein binding	5	12	4.5	2.165E-02	2.36E-01
GOTERM_MF_FAT	GO:0003779~actin binding	4	9	6.5	2.180E-02	2.23E-01
**Cluster 8**	**Enrichment Score: 1.6**					
GOTERM_CC_FAT	GO:0005739~mitochondrion	9	21	2.8	8.600E-03	3.53E-01
GOTERM_CC_FAT	GO:0044429~mitochondrial part	7	16	3.0	2.025E-02	4.03E-01
GOTERM_BP_FAT	GO:0006119~oxidative phosphorylation	4	9	6.0	2.658E-02	5.00E-01
GOTERM_CC_FAT	GO:0031966~mitochondrial membrane	5	12	3.7	3.916E-02	5.54E-01
GOTERM_CC_FAT	GO:0005740~mitochondrial envelope	5	12	3.4	4.839E-02	5.66E-01
