# Supplementary material for: Proteomic Profiling in Drosophila Reveals Potential Dube3a Regulation of the Actin Cytoskeleton and Neuronal Homeostasis
Source: PLoS One. 2013 Apr 23;8(4):e61952. doi: 10.1371/journal.pone.0061952 (PMC3633955; doi:10.1371/journal.pone.0061952)
Supplement: File S1 — This Microsoft PowerPoint file contains images of gels used for either direct comparison of experimental and control lanes or to excise a previously identified band for proteomic identification. The band numbers and gel numbers can be found in Table S1. (PPTX) [file pone.0061952.s004.pptx]

## Slide 1
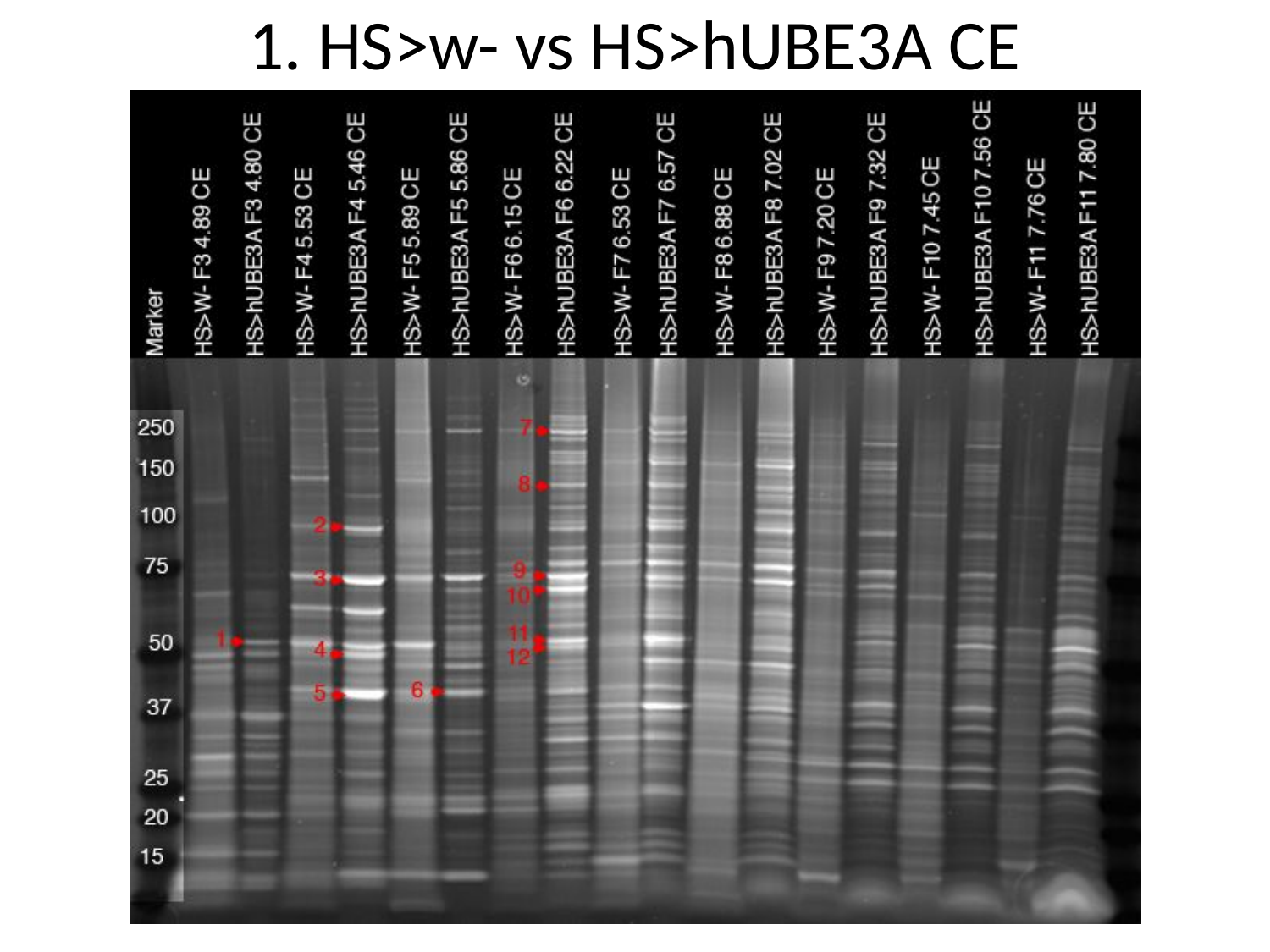

# 1. HS>w- vs HS>hUBE3A CE

## Slide 2
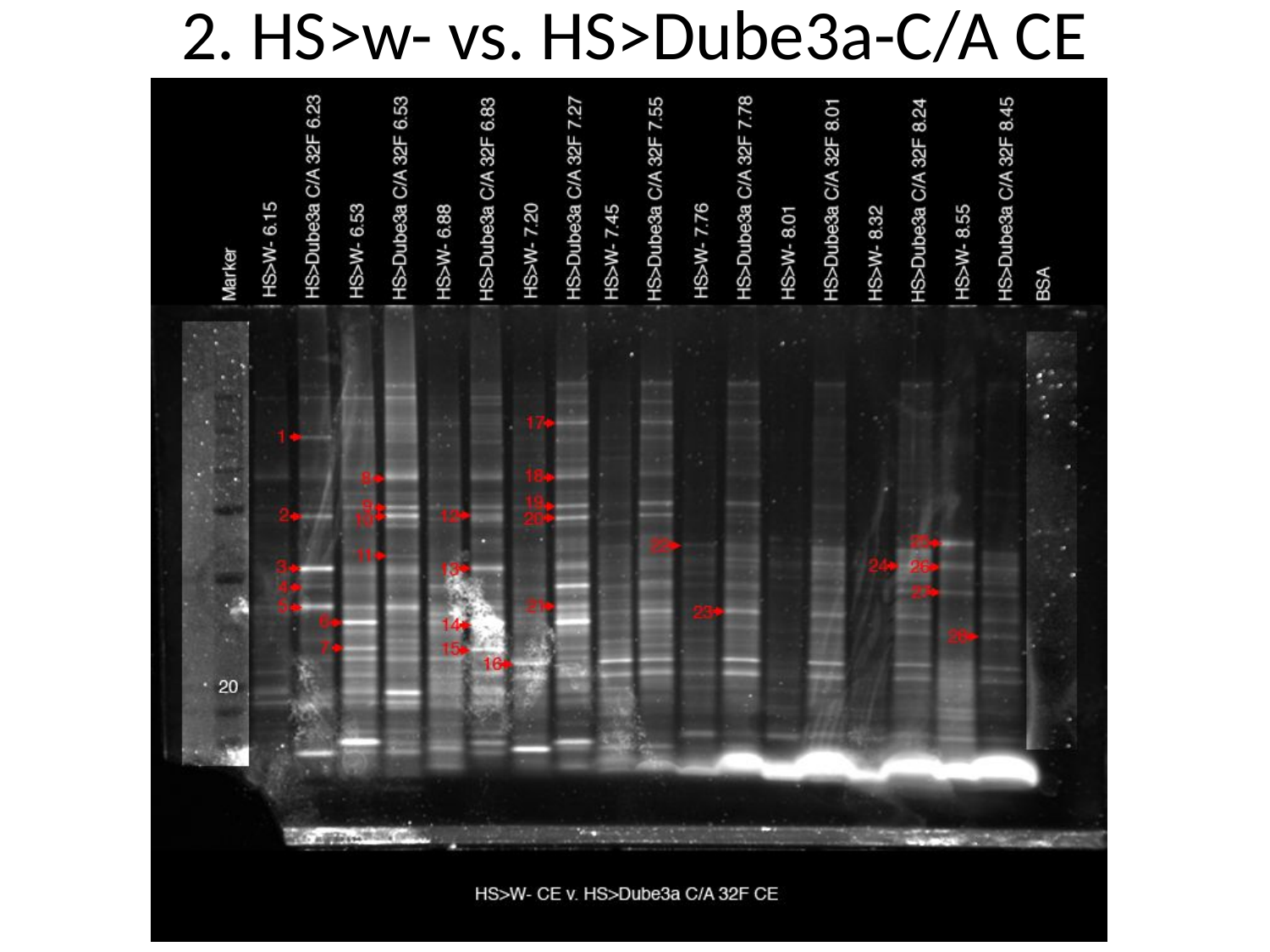

# 2. HS>w- vs. HS>Dube3a-C/A CE

## Slide 3
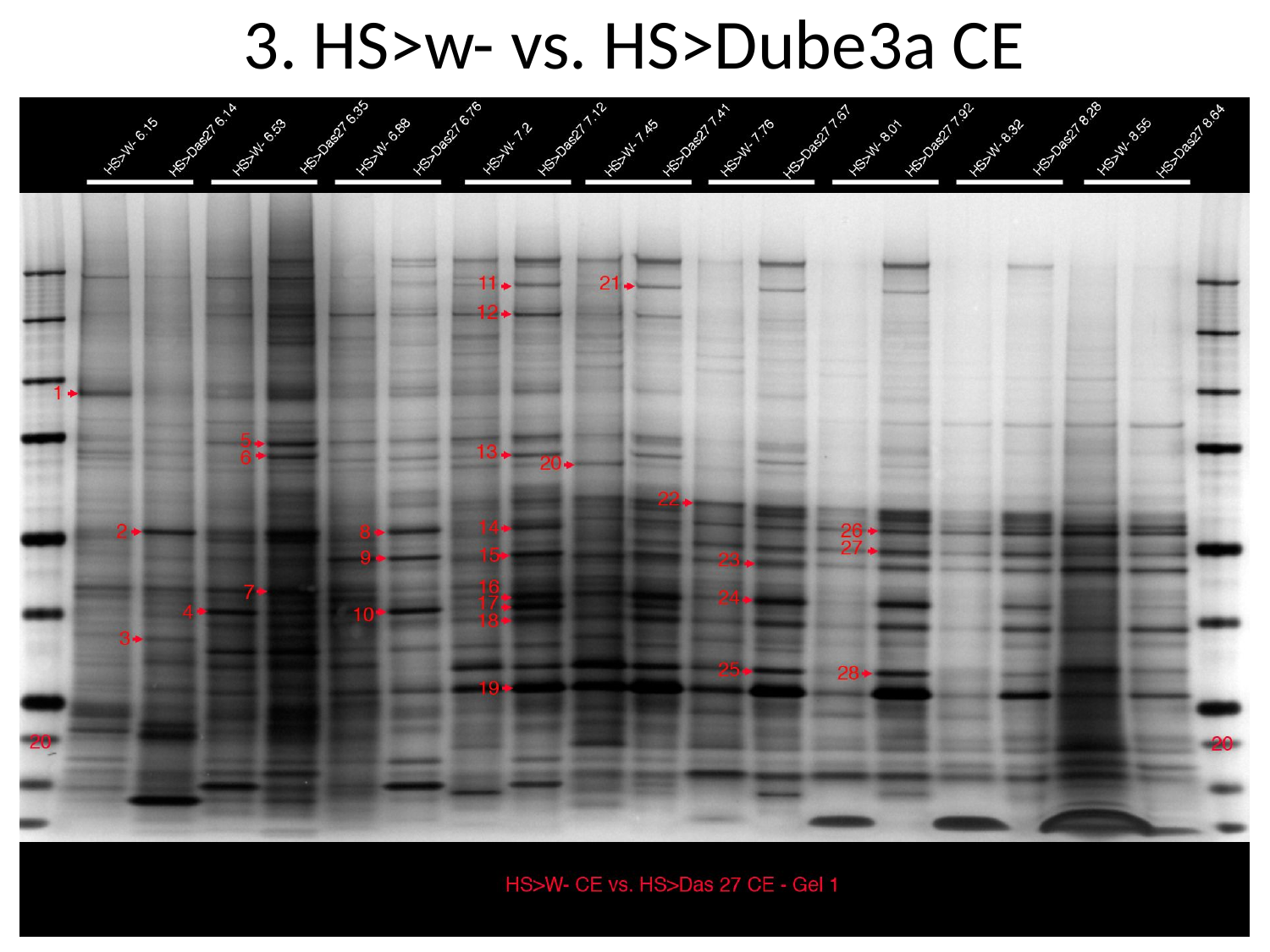

# 3. HS>w- vs. HS>Dube3a CE

## Slide 4
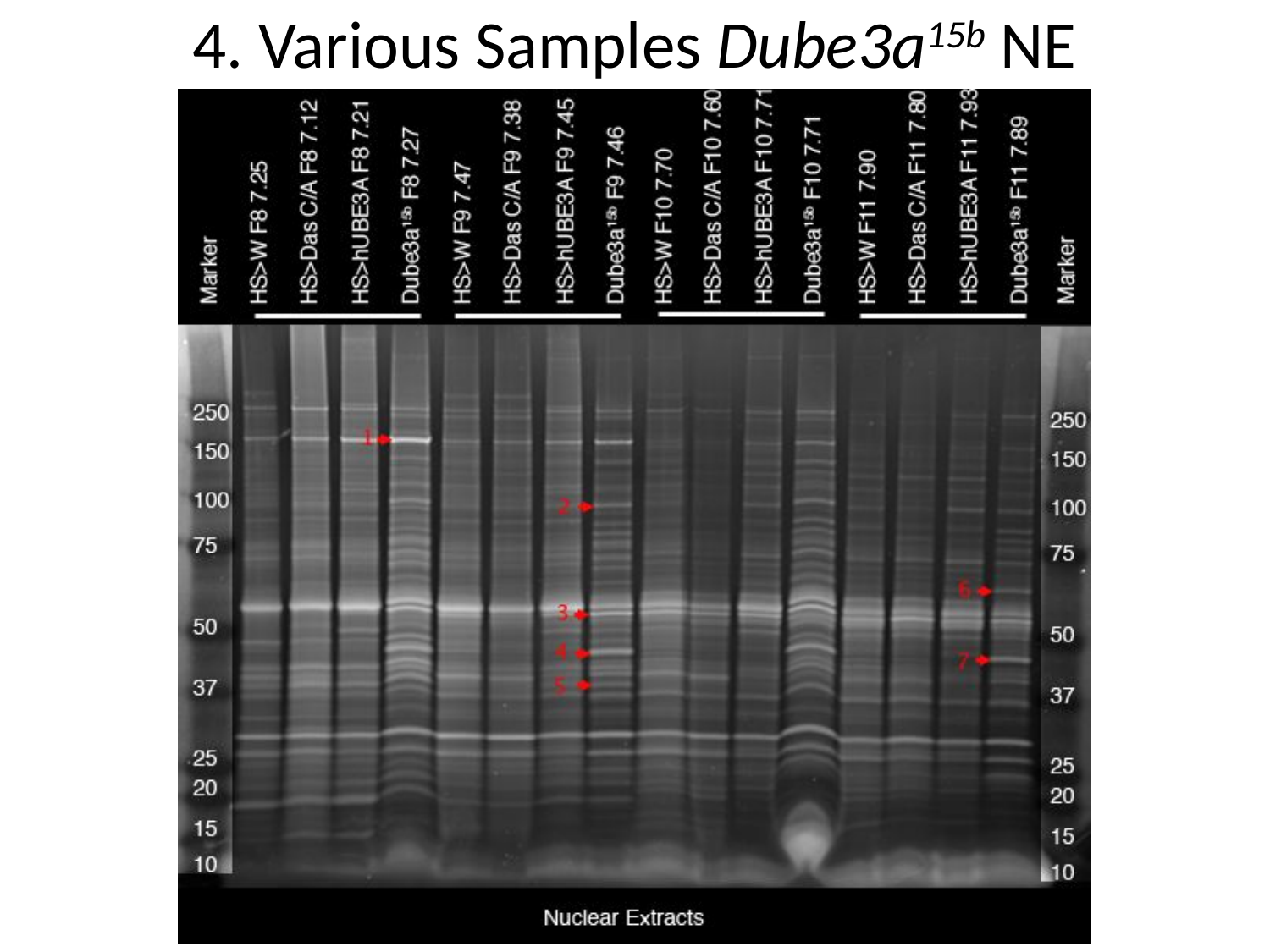

# 4. Various Samples Dube3a15b NE

## Slide 5
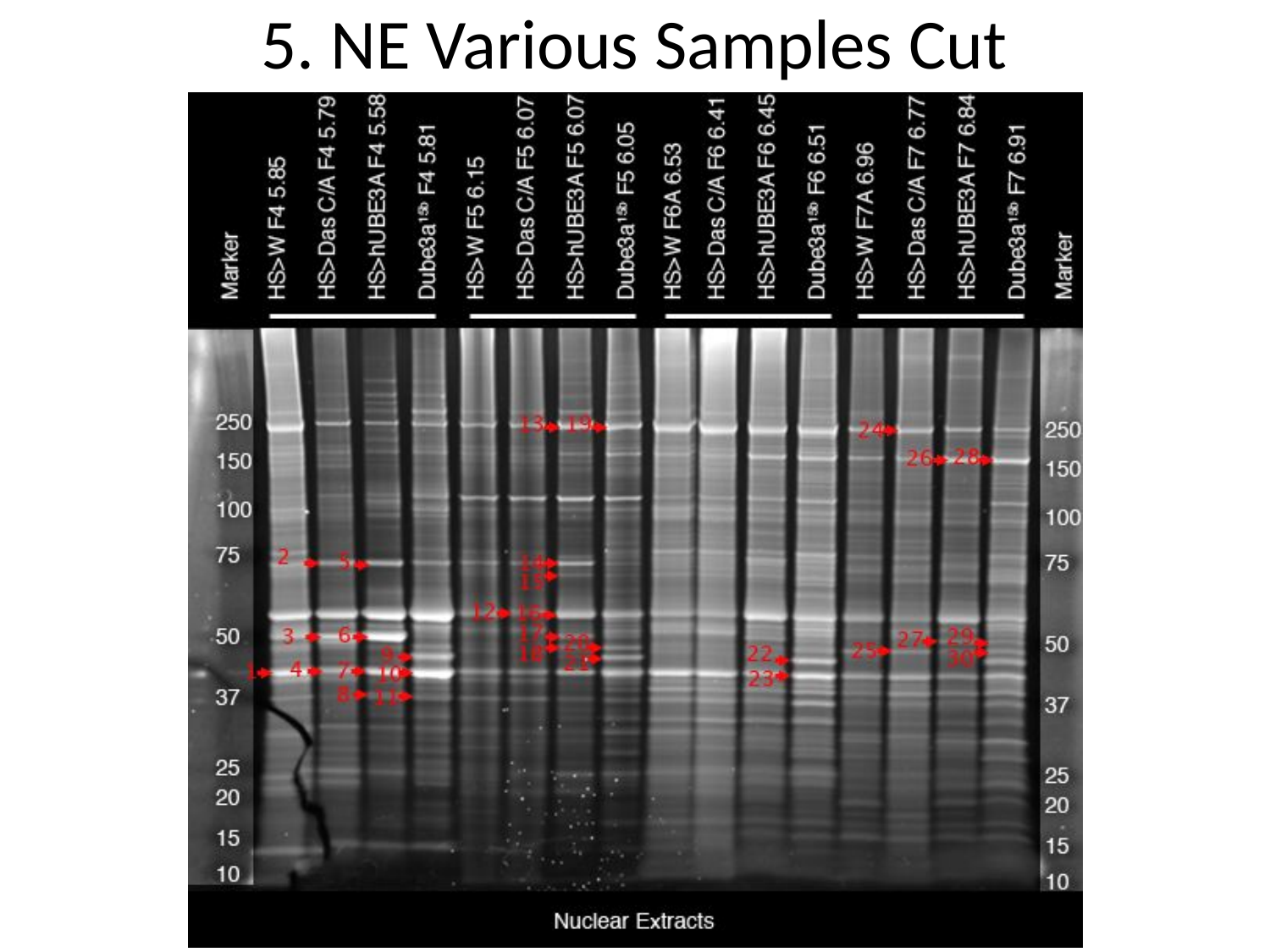

# 5. NE Various Samples Cut

## Slide 6
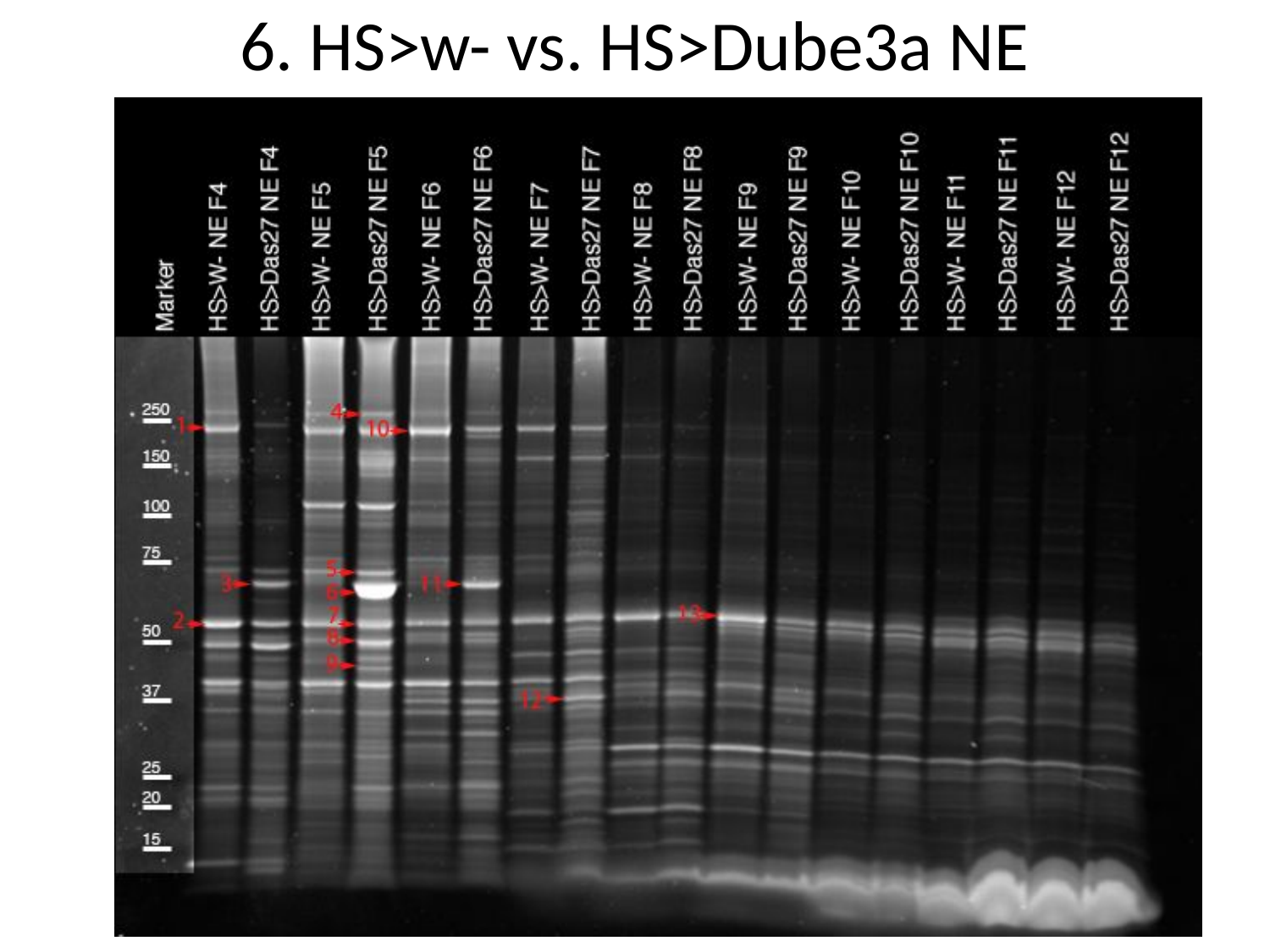

# 6. HS>w- vs. HS>Dube3a NE

## Slide 7
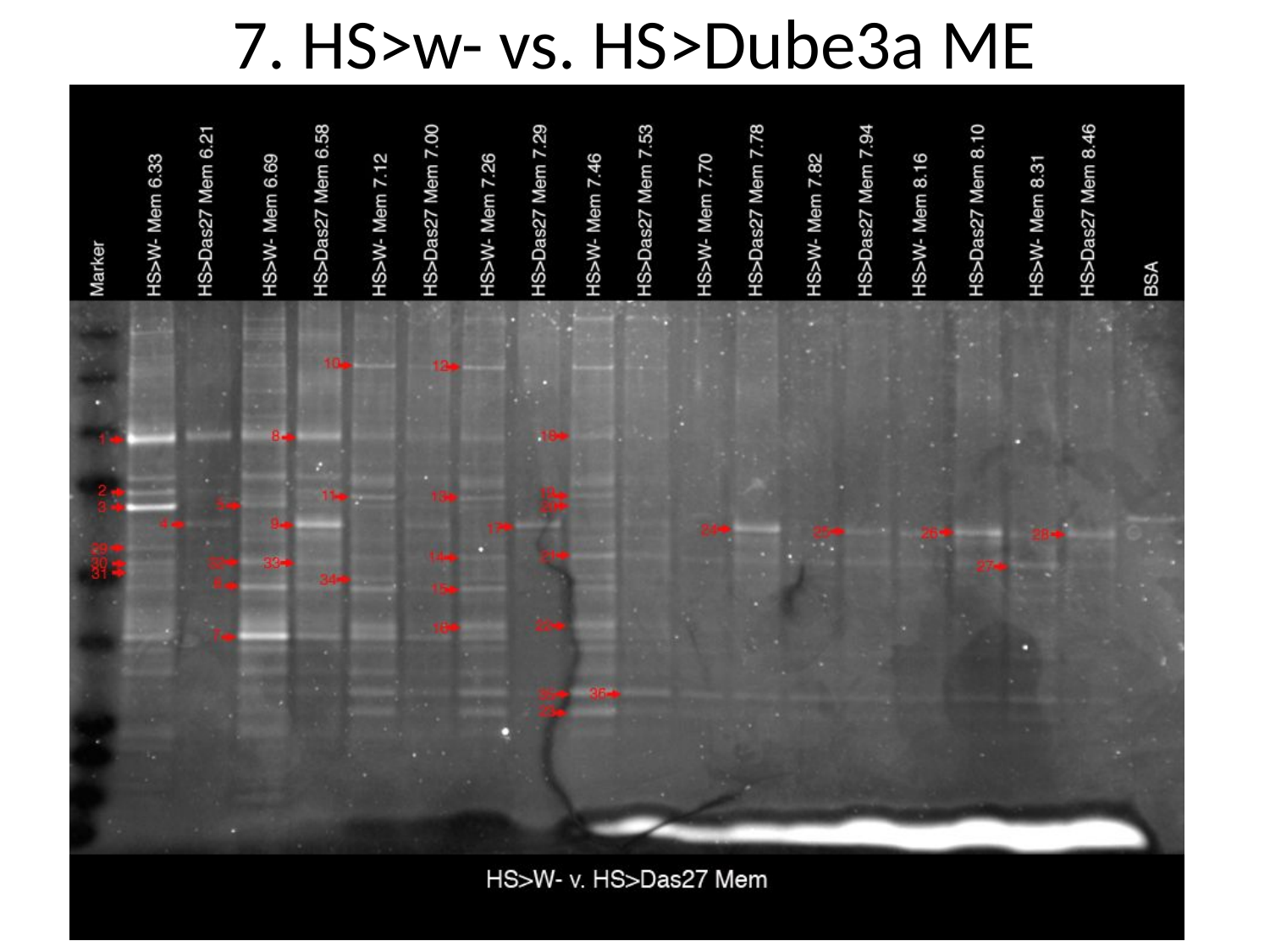

# 7. HS>w- vs. HS>Dube3a ME

## Slide 8
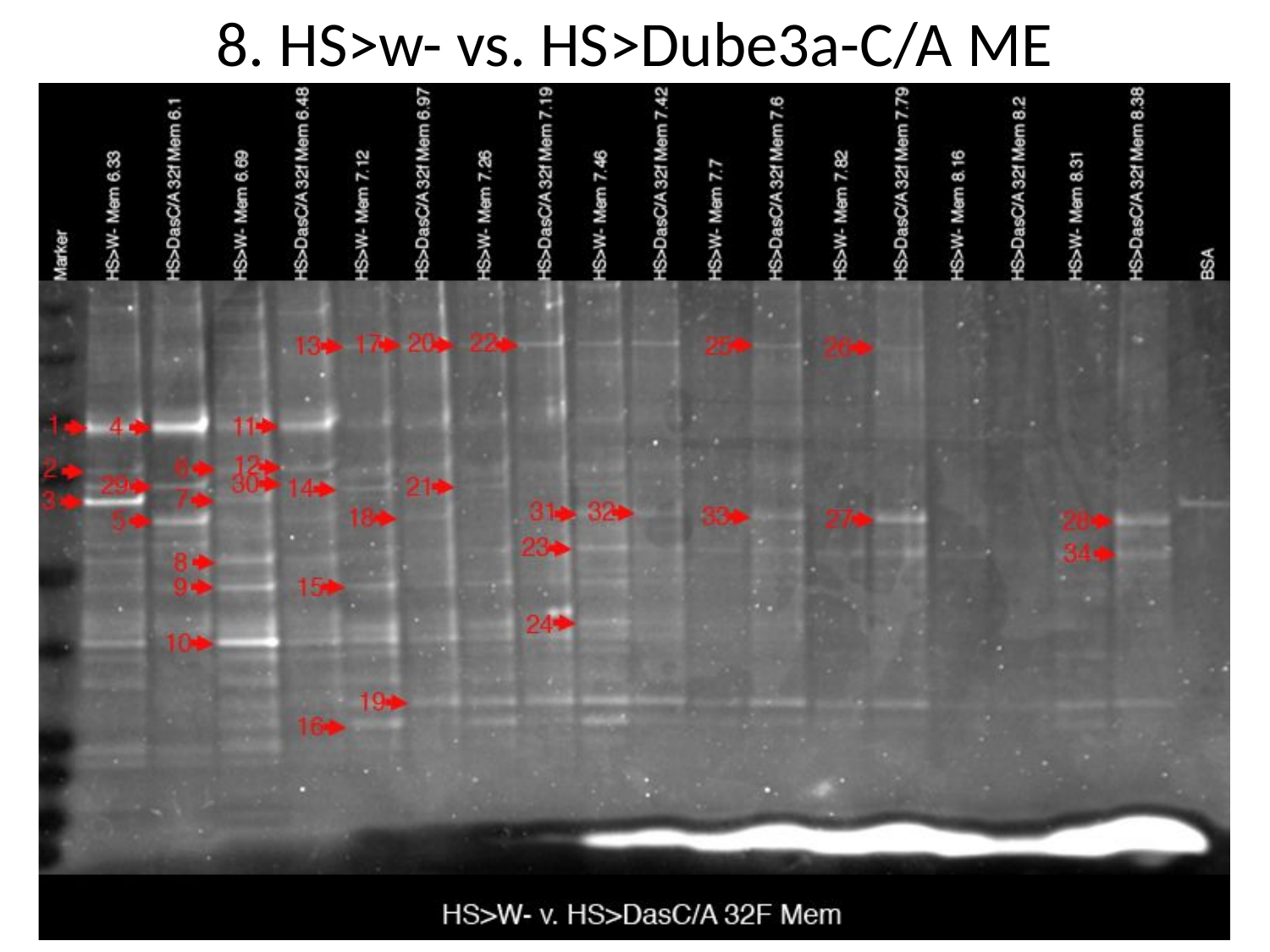

# 8. HS>w- vs. HS>Dube3a-C/A ME

## Slide 9
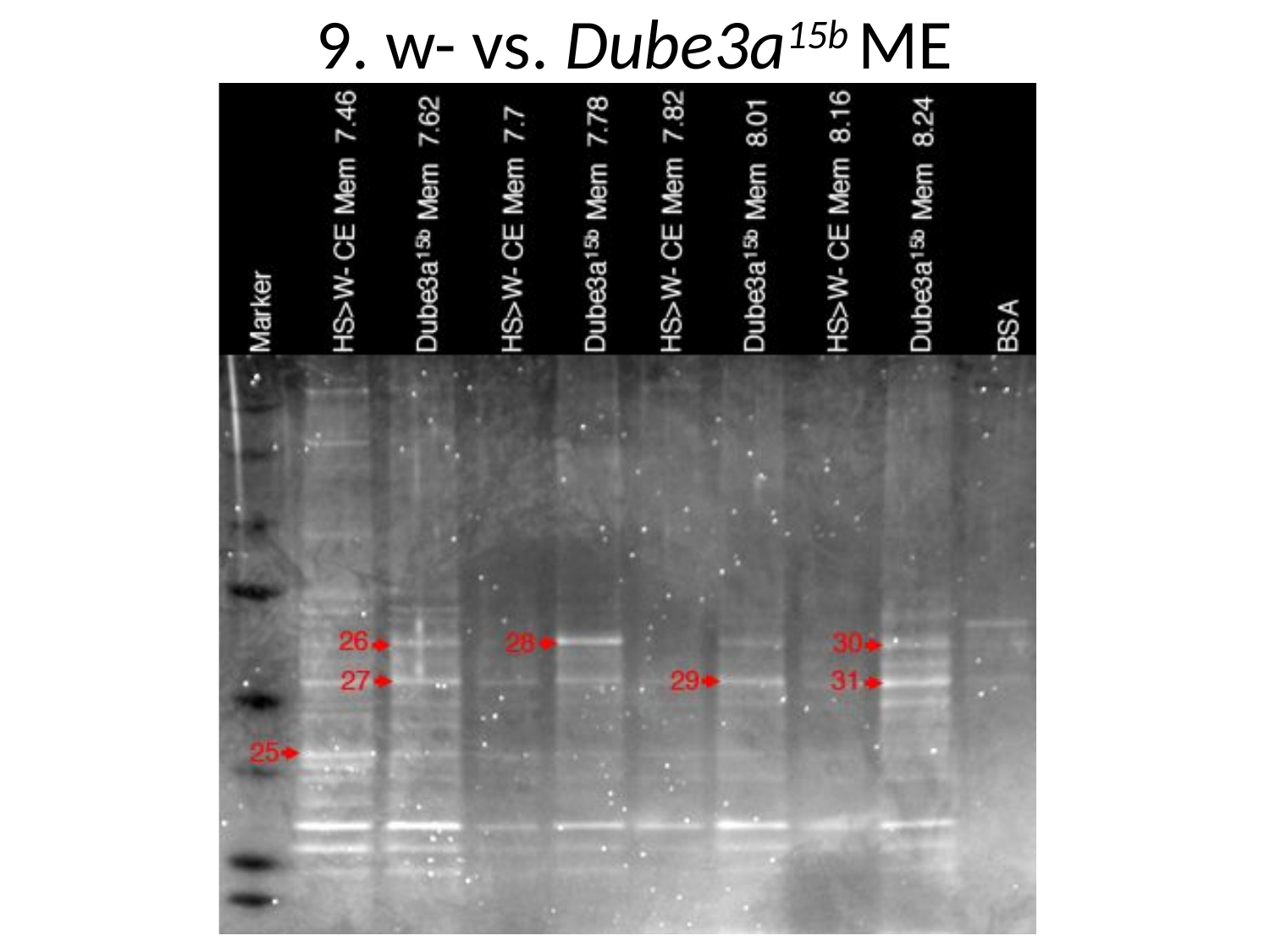

# 9. w- vs. Dube3a15b ME

## Slide 10
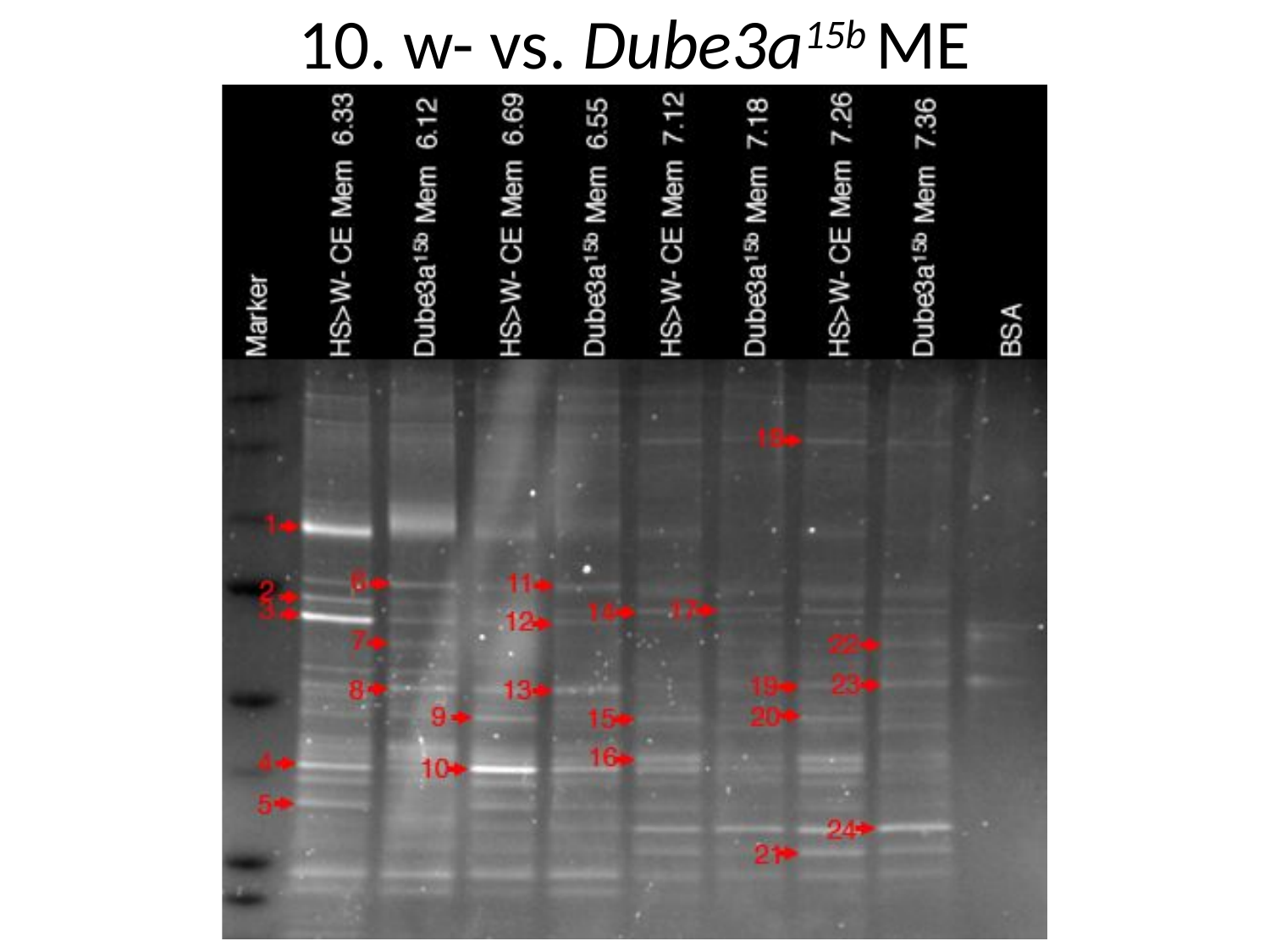

# 10. w- vs. Dube3a15b ME

## Slide 11
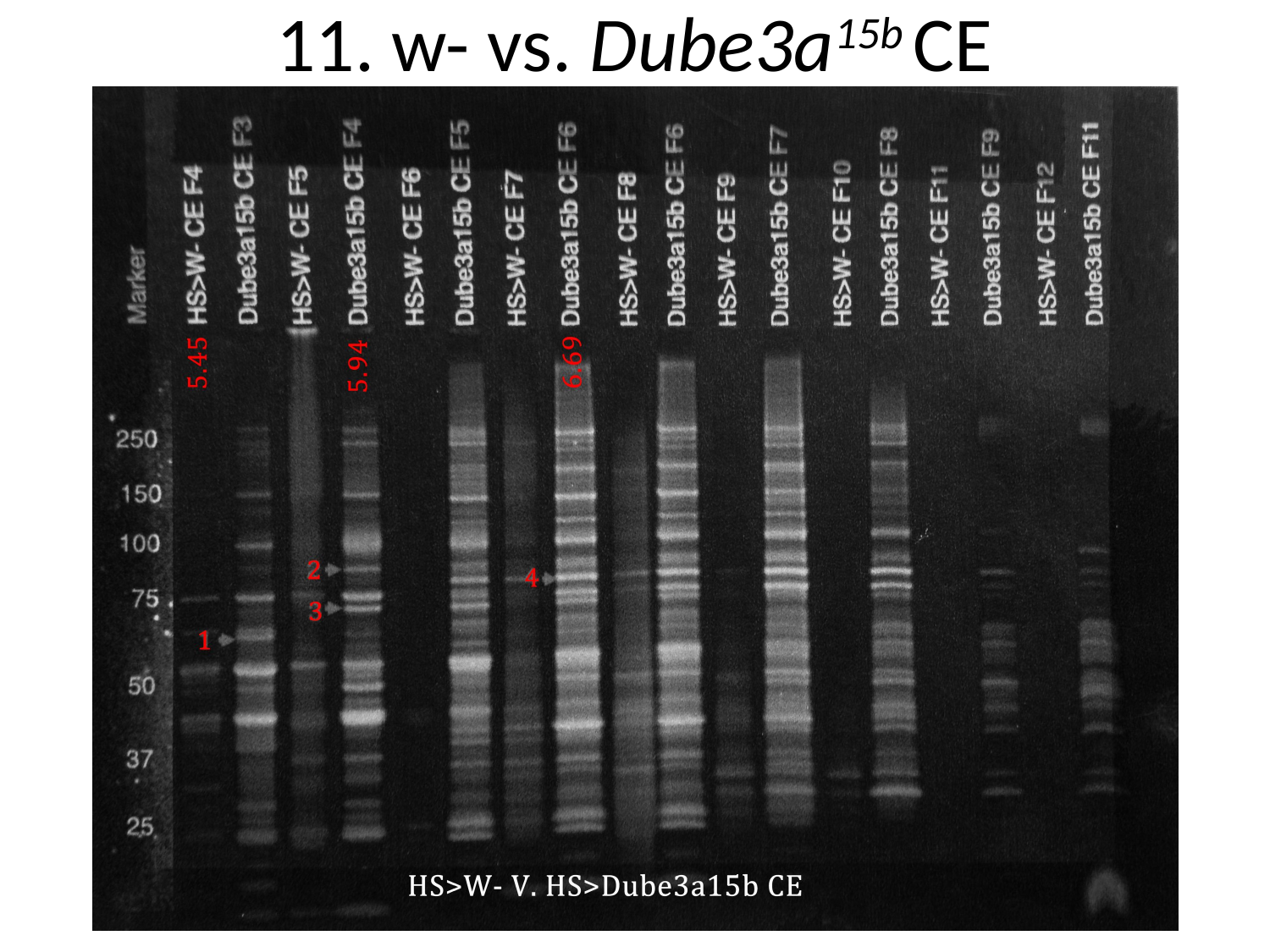

# 11. w- vs. Dube3a15b CE
